# Epstein-Barr viral product-containing exosomes promote fibrosis and nasopharyngeal carcinoma progression through activation of YAP1/FAPα signaling in fibroblasts

**DOI:** 10.1186/s13046-022-02456-5

**Published:** 2022-08-20

**Authors:** Po-Ju Lee, Yun-Hua Sui, Tzu-Tung Liu, Ngan-Ming Tsang, Chen-Han Huang, Ting-Yi Lin, Kai-Ping Chang, Shu-Chen Liu

**Affiliations:** 1 Department of Biomedical Sciences and Engineering, National Central University, 300, Zhongda Rd., Jhongli Dist, Taoyuan City, 32001 Taiwan; 2 Department of Radiation Oncology, Linkou Chang Gung Memorial Hospital, No.5, Fuxing St., Guishan Dist, Taoyuan City, 333 Taiwan; 3 Department of Radiation Oncology, China Medical University Hsinchu Hospital, No. 199, Sec. 1, Xinglong Rd.,Zhubei City, Hsinchu County, 302 Taiwan; 4 Department of Otolaryngology-Head & Neck Surgery, Chang Gung Memorial Hospital, No.5, Fuxing St., Guishan Dist, Taoyuan City, 333 Taiwan

**Keywords:** Epstein-Barr virus, EBV, Exosome, Nasopharyngeal carcinoma, NPC, Fibroblast, YAP1, FAPα, Fibrosis, Fibroblast activation protein alpha

## Abstract

**Background:**

The progression of nasopharyngeal carcinoma (NPC) is profoundly affected by Epstein-Barr virus (EBV) infection. However, the role of EBV in the intercommunication between NPC and surrounding stromal cells has yet to be explored.

**Methods:**

NPC biopsies were obtained for immunohistochemical (IHC) analyses. Clinical correlations between the expression of active YAP1/FAPα and the fibrotic response and between YAP1/FAPα and the density of cytotoxic CD8a^+^ T lymphocytes were determined. Survival times based on IHC scores were compared between groups using Kaplan-Meier survival and log-rank tests. Independent prognostic factors for metastasis/recurrence-free survival and overall survival were identified using univariate and multivariate Cox regression models. Fibroblasts were isolated from human nasopharyngeal biopsies. Exosomes were purified from culture supernatants of EBV^+^-positive NPC cells. The effects of EBV product-containing exosomes on fibroblast activation, fibrotic response, tumor growth, immune response, and correlations between the expression of featured genes were investigated using gel contraction assays, ELISAs, EdU incorporation assays, real-time impedance assays, RNA sequencing, immunostaining, 3D cancer spheroid coculture systems, and an NPC xenograft model.

**Results:**

NPC patients who developed metastasis had significantly higher levels of active YAP1 and FAPα in their tumor stroma, which was further correlated with tumor fibrosis and poorer metastasis-free survival. Exosomes released from EBV^+^-NPC cells contained abundant FAPα protein and EBV-encoded latent membrane protein 1. Viral product-containing exosomes markedly enhanced the fibrotic response and tumor growth in a mouse xenograft model. IHC analyses of human NPC and NPC xenografts revealed positive correlations between levels of active YAP1 and FAPα, YAP1 and the fibrotic response, and FAPα and the fibrotic response. Mechanistic studies showed that treatment of fibroblasts with viral product-containing exosomes promoted the characteristics of cancer-associated fibroblasts by stimulating YAP1 signaling and the production of the immunosuppressive cytokines IL8, CCL2, and IL6. Inhibition of YAP1 activation markedly reversed these exosome-mediated protumoral effects, resulting in reduced contractility, inactivation of YAP1 signaling, and decreased production of immunosuppressive cytokines in fibroblasts. Furthermore, fibroblasts stimulated with these viral product-containing exosomes promoted NPC resistance to T cell-mediated cytotoxicity within tumor spheroids. In NPC tissues, a significant negative correlation was found between YAP1/FAPα and the density of CD8a^+^ T lymphocytes with a granzyme B signature.

**Conclusion:**

EBV orchestrates interactions with the host and surrounding stroma by stimulating the functions of YAP1 and FAPα in fibroblasts through exosome cargos to create a more immunosuppressive, proinvasive microenvironment.

**Supplementary Information:**

The online version contains supplementary material available at 10.1186/s13046-022-02456-5.

## Background

Tumorigenesis involves constant communication between cancer cells and surrounding stromal cells. Among stromal cell types, the most abundant and perhaps most significant are cancer-associated fibroblasts (CAFs). CAFs express fibroblast activation protein-alpha (FAPα), smooth muscle actin alpha (α-SMA), PDGF receptor alpha (PDGFRα), PDGF receptor beta (PDGFRβ), and/or fibroblast specific protein 1 (FSP-1/S100A4) [1, 2], depending on the type of cancer. In nasopharyngeal carcinoma (NPC), a high density of CAFs predicts poorer survival [3, 4]. Moreover, CAFs together with NPC cells promote neoangiogenesis in a manner that depends on vascular endothelial growth factor (VEGF) and C-X-C motif chemokine ligand 12 (CXCL12) [5]. Exosomes derived from cancer cells or stromal cells may participate in creating a protumorigenic or antitumorigenic microenvironment in a context-dependent manner [6]. For example, uptake of tumor-derived exosomes by endothelial cells, particularly under hypoxic conditions, stimulates angiogenesis [7, 8], which may affect not only tumor growth but also metastasis [9]. In addition, exosomes can significantly impact the tumor microenvironment (TME) by regulating tumor immunity [10]. In NPC, Epstein-Barr virus (EBV) exhibits a type II latent infection in cancer cells [11] and establishes a persistent infection by escaping immune surveillance [12]. EBV-encoded latent protein 1 (LMP1) is the most important viral oncoprotein in NPC. LMP1 protein regulates the NF-κB and STAT3 signaling pathways through its C-terminal domain, thereby modulating the immune response in cancer cells [13, 14]. EBV itself and LMP1 protein not only direct the distribution of infiltrated lymphocytes but also modulate the expression of cytokines and cytokine receptors in host cells, manipulating cellular functions to benefit the infecting virus [15–18]. LMP1-mediated release of cytokines, chemokines and tumor-promoting factors, such as leukemia inhibitory factor (LIF), interleukin (IL)-6, transforming growth factor (TGF)-β, vascular endothelial growth factor (VEGF), fibroblast growth factor (FGF)-2, IL1, IL8, CXCL1 and matrix metalloproteinases (MMPs), can also modulate properties of the TME [11, 13, 19, 20]. Moreover, EBV-positive NPC cells release exosomes containing LMP1 and EBV-encoded microRNAs (miRNAs) [21–23]. EBV product-containing exosomes were shown to be transferred to uninfected cancer cells where they exert biological effects [21–23]. Importantly, LMP1 and EBV-encoded miRNA-containing exosomes have been found in serum and saliva samples of patients with EBV-positive NPC and are used as diagnostic markers [24, 25]. In addition to EBV genome-encoded products, which contribute to NPC pathogenesis, emerging evidence has demonstrated that host-stromal interactions also play a pivotal role in NPC malignancies [26–28]. However, the regulatory network underlying EBV-mediated remodeling of the NPC tumor stroma remains largely unexplored.

YAP1 signaling controls diverse cellular functions, with precise control of YAP1 localization and expression being crucial for accurate regulation [29–31]. Nuclear YAP1 is important in regulating cytoskeletal reorganization and mechanical transduction, and YAP1 was recently identified as a mechanoactivated coordinator of matrix-driven tissue fibrosis [32, 33].

In the current study, we investigated how EBV orchestrates interactions with the host and surrounding stromal fibroblasts within the NPC tumor microenvironment. We found that elevated levels of YAP1 and FAPα in stromal fibroblasts were significantly correlated with NPC tumor fibrosis and poorer metastasis-free survival. Notably, we demonstrated that treatment of primary fibroblasts with FAPα/EBV-LMP1-containing exosomes derived from NPC cells activated fibroblasts by stimulating the functions of YAP1 and FAPα, thereby enhancing fibrotic responses and tumor growth in mouse xenografts. In contrast, treatment of fibroblasts with selective YAP1 inhibitors significantly decreased these exosome-mediated effects. Importantly, fibroblasts stimulated with EBV-exosomes enhanced NPC resistance to T cell-mediated cytotoxicity within a 3D spheroid model. Immunohistochemical (IHC) analyses of human NPC biopsies showed that a higher density of YAP1 and FAPα in stromal fibroblasts was associated with a decreased abundance of tumor-infiltrating CD8a^+^ T lymphocytes with a granzyme B (GZMB) cytotoxic signature, implying that fibroblasts may also engage in modulating tumor immunity. These findings suggest that levels of YAP1 and FAPα might be useful as independent predictors of metastasis and that pharmaceutical interventions targeting YAP1 have the potential to break down the NPC tumor microenvironment and improve the therapeutic benefits.

## Methods

### Clinical samples

A total of 249 pretreatment, formalin-fixed paraffin-embedded (FFPE), EBV-positive NPC samples were collected from Linkou Chang Gung Memorial Hospital and subjected to IHC analysis. The study data were obtained through the medical records compiled by the doctors and the nursing staff and also the cancer registry, phone interviews and follow-up via correspondence with their homes or the household registration offices. A full course of therapy for NPC was defined as receiving external irradiation with a dosage of at least 68.4 Gy (Gray). The median dose of external radiotherapy was 72 Gy (68.4–82 Gy). The tumor node-metastasis (TNM) stage was defined according to the 2010 cancer staging system revised by the American Joint Committee on Cancer. All NPC tumors were histologically confirmed by pathologists. Patients were followed-up at 2- to 3-month intervals during the first 3 years after therapy and at 6-month intervals thereafter. The minimal follow-up period was 28 months. The time to local recurrence or distant metastasis was calculated using the date on which local recurrence or distant metastasis status was detected as the end point. Patients who died without occurrence of local recurrence or distant metastasis were censored in the analyses of local recurrence or distant metastasis free survival. Baseline characteristics of the YAP1 study population are summarized in Supplementary Table S1, and the characteristics of the FAPα study participants are summarized in Supplementary Table S2. Written informed consent was obtained from all participants prior to inclusion in the study.

### Cell culture

Primary fibroblasts were isolated from nasopharyngeal biopsies and maintained in DMEM (Hyclone) supplemented with 15% fetal bovine serum (FBS; Gibco) and 1% penicillin/streptomycin (Corning). A total of 5 primary fibroblast strains (Fibro#2, Fibro#10, Fibro#17, Fibro#18, and Fibro#20) were used in this study. The nasopharyngeal cell lines, HK1 and HK1EBV, as well as rAkata cells were grown in RPMI-1640 medium (Hyclone) supplemented with 10% FBS. NPC-TW06 cells [34] were grown in DMEM supplemented with 10% FBS. The authenticity of HK1, HK1EBV, NPC-TW06, and rAkata cells was confirmed using short tandem repeat (STR) profile analysis (conducted by the Bioresource Collection and Research Center, Taiwan).

### Animal studies

Non-obese diabetic (NOD)/severe-combined immunodeficient (SCID) mice were purchased from BioLASCO (Taiwan)*.* HK1EBV cells alone (1 × 10^6^ cells/100 μl) or together with primary fibroblasts (10^5^ cells/100 μl), with or without pretreatment with exosomes (15 μg/ml) for 3 hours, were subcutaneously injected into the thighs of 7-week-old male NOD/SCID mice. Tumor growth was evaluated by measuring tumor size with a caliper twice a week. The tumor volume was estimated by the formula: volume (mm^3^) = (L × W^2^) / 2, where L and W are the length and width, respectively. Mice were maintained under specific pathogen-free conditions and were sacrificed when tumor volumes reached approximately 1 cm^3^, at which point tumor tissues were harvested for IHC analyses. All animal experiments were conducted according to accepted principles of laboratory animal care and were approved by the animal committees of National Central University, Taoyuan, Taiwan.

### RNA sequencing

Total RNA from control (untreated) primary fibroblasts (Fibro#2) and fibroblasts treated with HK1EBV cell-derived exosomes (10 μg/ml) for 3 or 6 hours were extracted using an RNeasy Mini kit (Qiagen). cDNA libraries were constructed and sequenced on the Illumina NovaSeq platform. The cutoff value for differentially expressed genes (DEG) was defined as absolute log2 fold change > 0.5 (equal to a 1.42-fold change in the original scaling) and *p* < 0.05. Functional analyses, including Gene Set Variation Analysis (GSVA) [35] and KEGG pathway enrichment analysis, were performed using R package clusterProfiler [36], GSEA [37] and Ingenuity Pathway Analysis software (QIAGEN) based on statistical significance and DEG values.

### Collagen gel contraction assay

Fibroblast cell suspension was mixed with pH-adjusted collagen solution (Advanced Biomatrix) at a final collagen concentration of 1.98 mg/ml. A 500-μl sample of this cell-collagen mixture containing 3 × 10^4^ cells was seeded into wells pre-coated with bovine serum albumin (BSA) and incubated at 37 °C for 90 minutes until the gel solidified. Polymerized cell-collagen mixtures were then overlaid with 500 μl of DMEM supplemented with 5% FBS plus exosomes or inhibitors. After a 1-hour incubation, the edge of the gel was carefully dislodged with a 20-μl tip, and the area of gel contraction was measured using ImageJ software. All contractility experiments were conducted in triplicate, and at least three independent experiments were conducted.

### IHC analysis of FFPE tumor tissue sections

Pretreatment NPC FFPE biopsy sections were collected for examination of the expression of relevant proteins. Primary antibodies against the following proteins were used for IHC analyses: human FAPα (Abcam, ab207178), YAP1 (Cell Signaling Technology, #14074), active YAP1 (Abcam, ab205270), PDGFRα/β (Abcam, ab32570), CD8a (Cell Signaling Technology, #85336), and granzyme B (Cell Signaling Technology, #46890). The fibrotic response of the tumor was evaluated using trichrome staining (ScyTek). The IHC staining score and degree of fibrosis were defined according to staining intensity (“−”, “+”, “++” or “+++”) and extent (positive percentage relative to total area investigated). All biopsies were histologically confirmed by pathologists.

### Western blot

Cells and exosomes were lysed in NP40 lysis buffer containing a protease inhibitor cocktail (Roche). Equal amounts of protein were loaded and separated by sodium dodecyl sulfate-polyacrylamide electrophoresis (SDS-PAGE) and transferred to polyvinylidene fluoride membranes (GE Healthcare Life Science). The membranes were washed briefly with 0.1% Tween-20 in TBS (TBS-T), blocked with 5% BSA in TBS-T, and probed with specific primary antibodies at 4 °C overnight. After serial washes with TBS-T, the blots were incubated with horseradish peroxidase (HRP)-conjugated secondary antibody (Millipore), followed by development with chemiluminescence reagents.

### Isolation of exosomes

EBV-positive HK1EBV, rAkata, or HK1 cells were cultured overnight in complete RPMI-1640 at a density of 2 × 10^6^ cells/ml after which the medium was replaced with 10 ml of RPMI-1640 medium containing 1% EV-depleted FBS and cells were cultured for an additional 48 hours. The cell-conditioned supernatant was collected for total exosome isolation using ExoQuick-TC according the manufacturer’s instructions (System Biosciences). Isolated exosome pellets were lysed in 1% NP-40 lysis buffer for Western blot analysis or resuspended in an appropriate amount of PBS and stored at − 80 °C until use.

### TEM analysis of exosomes

A mixture of freshly prepared exosomes and magnetic beads (Invitrogen Dynabeads M^−^ 270 Epoxy) conjugated to anti-CD9 antibody (BD Pharmingen, 555,370) was incubated for 30 minutes and then centrifuged for 1 minute, yielding exosome-captured magnetic beads. The precipitated beads were blocked with 1% BSA/PBS, washed with PBS, and resuspended in PBS. Five microliters of this captured exosome solution was loaded onto a 200-mesh copper grid (TED PELLA INC), incubated for 8 minutes at room temperature, and washed with distilled water to remove excess sample. For negative staining, 1.5% (w/v) phosphotungstic acid hydrate solution (Merck, Darmstadt, Germany) was applied to the copper grid for 3 seconds followed by washing with distilled water. Exosomes were examined under a transmission electron microscope operating at 200 kV (JEM2000FX, JEOL).

### Exosomal stimulation of primary fibroblasts

Human fibroblasts were cultured in fresh DMEM supplemented with 3% FBS for 16 hours, and then treated with 10 μg/ml exosomes at 37 °C or varying durations, as indicated. Uptake of exosomes was evaluated by imaging analysis using ExoGlow (green)-labeled exosomes (System Biosciences) or detection of exosome-specific markers.

### Preparation of conditioned medium

Primary fibroblasts were plated at a density of 1 × 10^5^ cells/well in 6-well plates and grown for 1 day. The culture medium was replaced with DMEM supplemented with 3% FBS and grown at 37 °C for an additional 16 hours. Cells were then treated with exosomes or as described in the text. Cell-free culture supernatants were collected and centrifuged at 2000 rpm, 4 °C for 10 minutes. Conditioned medium was stored at − 80 °C until use. Verteporfin (1 μM) or saracatinib (20 μM) was added to the growth medium of fibroblasts 2 hours prior to exosome (10 μg/ml) treatment.

### Real-time monitoring of cellular responses using the xCelligence system

The xCelligence real-time analyzer (ACEA Biosciences) was utilized to monitor the growth and survival of cells based on measurements of cell impedance. Prior to cell seeding, the background impedance of each E-plate was determined by loading 50 μl/well of culture medium. Cells (3 × 10^3^ cells/well in 100 μl) were then plated and allowed to grow at 37 °C. Impedance was recorded every 10 minutes for 3 to 5 days. Cytokines [IL6 (20 ng/ml; R&D Systems), IL8 (50 ng/ml; R&D Systems), CCL2 (100 ng/ml; R&D Systems)], cytokine-specific neutralizing antibodies [IL6 (0.5 μg/ml; Abcam, Ab9324), IL8 (0.5 μg/ml; Cell Signaling, #94407), and CCL2 (0.5 μg/ml; Invitrogen, MA5–17040)], the corresponding IgG isotype control antibodies (0.5 μg/ml) (Croyez Bioscience), inhibitors (verteporfin and saracatinib), or conditioned medium was added at the indicated time points. All experiments were performed in quadruplicate.

### 5-Ethynyl-2-deoxyuridine (EdU) incorporation assay

DNA synthesis in cells was evaluated using a Click-iT EdU Alexa Fluor 488 EdU Incorporation kit (Invitrogen) according to the manufacturer’s protocol. Briefly, NPC cells were treated with conditioned medium or cytokines for 10 hours, followed by EdU (10 μM) labeling for 4 hours. Cells were then fixed and counterstained with Hoechst 33342 DNA dye. The number of EdU-positive cells was determined using cellSens Software (Olympus).

### Live-cell imaging

For live-cell imaging of the actin cytoskeleton, human primary fibroblasts were infected with adenovirus containing LifeAct-RFP (Ibidi) at a pre-optimized multiplicity of infection (MOI) according to the manufacturer’s protocol. For selection of stable clones, puromycin (2 μg/ml) (Invitrogen) was added to culture medium 48 hours after infection. Time-lapse experiments were performed in a microscope chamber maintained at 37°Cand 5% CO2 (Olympus IX830). Phase-contrast and fluorescent images were simultaneously acquired every 2 minutes, and movies were obtained using CellSens imaging software (Olympus).

### Quantitative real-time reverse transcription-polymerase chain reaction (qRT-PCR) analysis

An optimal amount of total RNA was reverse transcribed into cDNA using a first strand cDNA synthesis kit (Roche) according to the manufacturer’s instructions. Diluted cDNAs were mixed with Master mix reagents (Bio-Rad), and PCR was performed on a LightCycler 96 Instrument (Roche) following the manufacturer’s protocol. Primer information is summarized in Supplementary Table S3. All assays were repeated in duplicate.

### T cell cytotoxicity assay of NPC spheroids

Human peripheral blood-derived T cells were expanded and activated by using recombinant IL-2 (30 U/ml) (R&D Systems) and Dynabeads magnetic beads coated with anti-CD3 and anti-CD28 antibodies (Gibco). A 3-dimensional spheroid coculture model was established for tumor killing assays by activated T cells. Cancer spheroids were generated according to the manufacturer’s instructions (STEMCELL Technologies). Briefly, NPC-TW06-GFP cells (9 × 10^5^ cells/well) alone or mixed with human fibroblasts (2 × 10^5^ cells/well) were added to the wells containing microwells of a 24-well AggreWell plate (STEMCELL Technologies) and centrifuged to evenly distribute cells in the microwells. Spheroids were formed after 24 hours of incubation. Four-day activated T cells were then added to spheroid cultures with an effector/target ratio of 3:1 and cultured for another 24 hours. T cell-mediated tumor killing was evaluated via microscopic examination (Olympus IX 83), and the integrated GFP intensity of spheroids was calculated using ImageJ software.

### Reagents and antibodies

Stock solutions of saracatinib (LC Laboratories) and verteporfin (AdooQ Bioscience) were prepared by dissolving in DMSO at final concentrations of 369 and 69 mM, respectively. Unless otherwise indicated, saracatinib and verteporfin were used at final concentrations of 20 μM and 1 μM, respectively, in all drug treatment experiments. The human recombinant cytokines, IL6 (20 ng/ml; R&D Systems), IL8 (50 ng/ml; R&D Systems) and CCL2 (100 ng/ml; R&D Systems), were used to investigate effects of cytokines on cell proliferation and activation of signaling pathways in NPC cells. Human ELISA kits (R&D Systems) were used to detect levels of IL6, IL8, and CCL2 secreted into the culture medium of fibroblasts. Treatment of Brefeldin A (BFA; BD Biosciences, 555,029) was used to increase the detectability of intracellular cytokines by Western blotting. The indicated antibodies against the following proteins were used for Western blotting: calnexin (Cell Signaling, #2679), phospho-YAP (Cell Signaling, #13008), YAP (Cell Signaling, #14074), CYR61 (Cell Signaling, #14479), CTGF (Cell Signaling, #86641), IGFBP3 (Cell Signaling, #25864), PDGFRα/β (Abcam, ab32570), α-SMA (Abcam, ab124964), phospho-mTOR (Cell Signaling, #2971), phospho-p70S6K1 (Cell Signaling, #9234.), phospho-MEK1/2 (Cell Signaling, #9121), MEK1/2 (Cell Signaling, #4694), phospho-p38 MAPK (Cell Signaling, #4511), p38 MAPK (Cell Signaling, #9212), phospho-ERK1/2 (Cell Signaling, #4370), and ERK1/2 (Cell Signaling, #4695), CD9 (Abcam, ab92726), GAPDH (Abcam, ab9484), active YAP1 (Abcam, ab205270), mTOR (Abcam, ab2732), p70S6K1 (Abcam, ab32529), HSP70 (System Biosciences, EXOAB-HSP70A-1), β-actin (Sigma, A5060), α-tubulin (Santa Cruz Biotechnology, sc32293), and EBV-encoded LMP1 monoclonal antibody (S12), purified from hybridoma culture supernatants. Uncut western blots for all figures were summarized in Supplementary Fig. S1. Primary antibodies against the following proteins were used for immunocytochemistry: FAPα (Santa Cruz Biotechnology, sc65398,), active YAP1 (Abcam, ab205270), CYR61 (Cell Signaling, #14479), IGFBP3 (Cell Signaling, #64143), and CTGF (Cell Signaling, #86641).

### Statistics

Statistical analyses were performed using SPSS 16.0 (SPSS) or GraphPad Prism 9 (GraphPad Software). The statistical significance of immunoreactivities in human biopsies was assessed using the chi-square test. Spearman’s rank correlation coefficient was used to evaluate correlations between IHC results (staining intensity × percentage). Kaplan-Meier survival and log-rank tests were used to compare survival times between groups based on IHC scores. Univariate and multivariate Cox regression models were used to identify independent prognostic factors for metastasis-free, recurrence-free, and overall survival. Variables tested in univariate and multivariate analyses included YAP1 or FAPα score, treatment days, biologically effective dose, age, gender, T stage, N stage, chemotherapy, comorbidity, smoking status, betel quid, and alcohol consumption. All statistical tests were two-sided, and *p*-values < 0.05 were considered statistically significant.

## Results

### Higher nuclear levels of YAP1 and FAPα expression in fibroblasts are correlated with tissue fibrosis and poorer prognosis in NPC

**Fig. 1:**
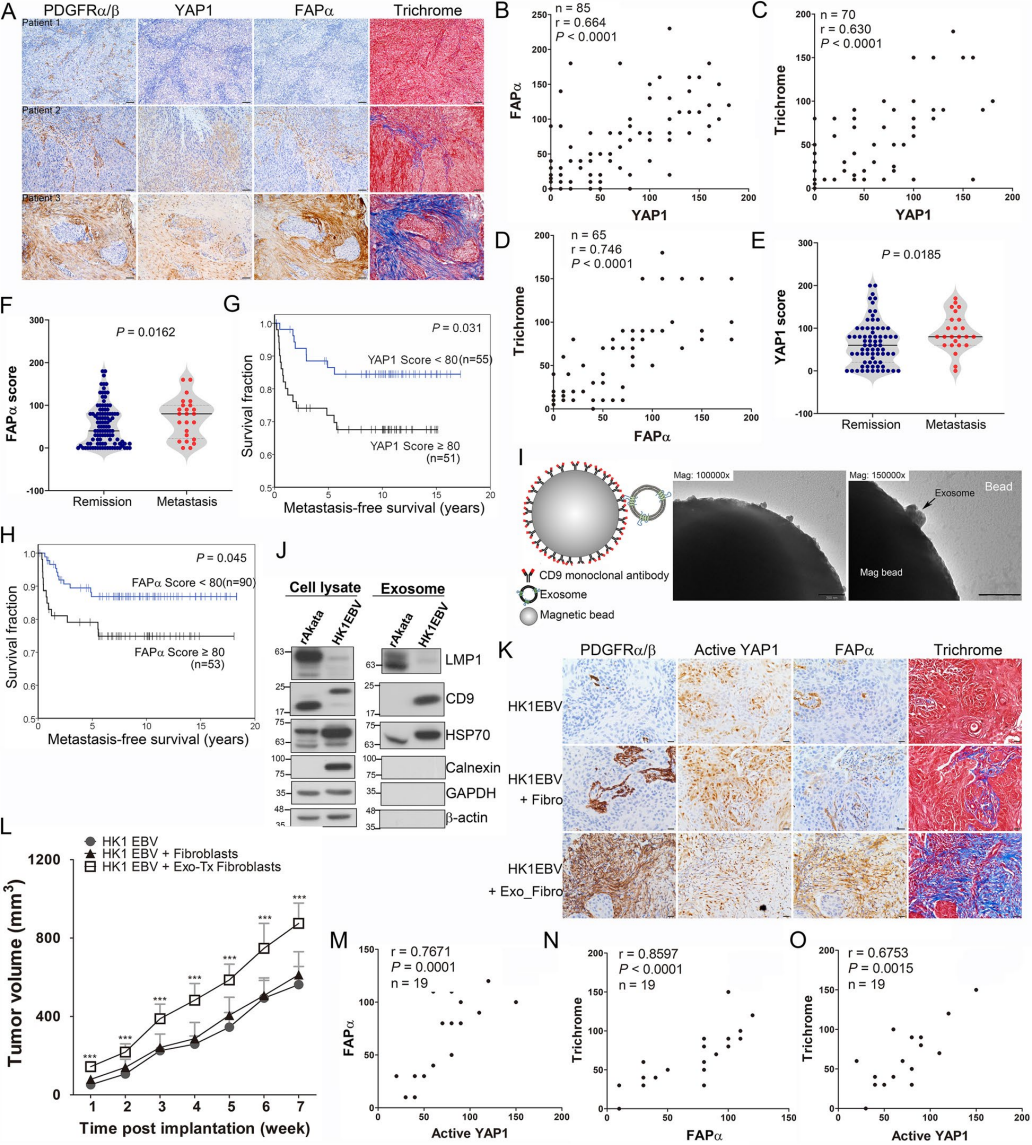
Increased active YAP1 and FAPα levels in fibroblasts are associated with tissue fibrosis and cancer progression in NPC. **A** Representative IHC images of PDGFRα/β, YAP1 and FAPα, and trichrome staining in NPC tumor fibroblasts. Immunoreactivity was analyzed in FFPE consecutive NPC tissue sections. Scale bar, 50 μm. **B–D** Correlation analysis of nuclear YAP1 and FAPα expression (**B**), nuclear YAP1 and trichrome signals (**C**), and FAPα and trichrome signals (**D**) based on IHC scores in tumor sample fibroblasts (Spearman’s correlation test). **E–F** Statistical analysis of nuclear YAP1 (**E**) and FAPα (**F**) expression in stromal fibroblasts of NPC tumors obtained from patients with complete tumor remission or distant metastasis after treatment (chi-square test). **G–H** Kaplan–Meier metastasis-free survival curves of NPC patients based on nuclear YAP1 levels (**G**) or FAPα levels (**H**). **I** TEM analysis of HK1EBV cell-derived exosomes. Scale bar, 200 nm. **J** Western blot analysis of exosomal proteins. Cell and exosome lysates extracted from rAkata cells, which were used as EBV-positive controls. Cell lysates and exosomes derived from 2-day–cultured supernatants of HK1EBV cells or rAkata cells. **K** Representative IHC images of PDGFRα/β, active YAP1 and FAPα, and trichrome staining in NPC xenografts. Scale bar, 20 μm. **L** Tumor growth in NPC xenografts. HK1EBV cells, HK1EBV cells together with fibroblasts, or HK1EBV cells together with exosome-treated fibroblasts were subcutaneously injected into the legs of mice (*n* = 8–9 mice/group). Data are presented as means and SEMs (***p < 0.001, compared with the other two groups; paired t test). **M–O** Correlation analyses based on IHC scores in NPC xenografts (Spearman’s correlation test)

We previously showed that nuclear YAP1 (the activated form of YAP1) expression in NPC cells is associated with NPC metastatic behavior [38]. Intriguingly, our current findings revealed that nuclear YAP1 is also detected in stromal fibroblasts in certain NPC cases. YAP1 was reported to regulate mechanotransduction [32, 33, 39–43], indicating that the activation of YAP1 may be associated with the acquisition of the CAF phenotype. To address this issue, we first examined whether the expression levels of YAP1 and FAPα are correlated in stromal fibroblasts in NPC biopsies. Our IHC analyses showed that high YAP1 expression in the stromal compartment overlapped with enhanced FAPα immunoreactivity that was accompanied by a fibrotic response, as evaluated by trichrome staining (Fig. 1A). Stromal fibroblast-like cells were identified by spindle-like morphology and expression of a CAF marker, PDGFRα/β (Supplementary Fig. S2). Fibroblasts that expressed low levels of YAP1 exhibited reduced FAPα expression and a decreased extent of fibrosis (Fig. 1A; patient 1). These results suggest that YAP1 might be a critical regulator of the fibrotic response in NPC. Additional analyses revealed marked correlations between the expression of nuclear YAP1 and FAPα (*p* < 0.0001, *r =* 0.664) (Fig. 1B), nuclear YAP1 and fibrotic intensity (p < 0.0001, *r =* 0.63) (Fig. 1C), and FAPα and fibrotic intensity (p < 0.0001, *r =* 0.746) (Fig. 1D) in stromal fibroblasts. IHC analyses further showed stronger nuclear YAP1 and FAPα immunoreactivity in stromal fibroblasts of tumors obtained from the NPC patients diagnosed with distant metastasis compared with that in the patients with complete tumor remission after therapy (*p =* 0.0185 and 0.0162 for YAP1 and FAPα, respectively) (Fig. 1E and F). Furthermore, the NPC patients with elevated nuclear YAP1 expression in fibroblasts had significantly poorer metastasis-free survival than the patients with lower YAP1 expression (*p =* 0.031) (Fig. 1G). Similarly, higher levels of FAPα were found to correlate with poorer metastasis-free survival (*p =* 0.045) (Fig. 1H). The clinicopathological characteristics of the YAP1 and FAPα study participants are summarized in Supplementary Tables S1 and S2, respectively. Multivariate Cox regression analyses indicated that a higher level of YAP1 (YAP1 score ≥ 80) in fibroblasts was an independent prognostic factor for metastasis-free survival (*p =* 0.001, hazard ratio [HR] = 4.834, 95% confidence interval [CI] = 1.857–12.579) (Table 1), recurrence-free survival (*p =* 0.011, HR = 3.841, 95% CI = 1.356–10.883) (Table 2), and overall survival (*p =* 0.011, HR = 2.402, 95% CI = 1.218–4.737) (Table 3) in NPC patients. Multivariate Cox regression analyses also indicated that higher FAPα expression (FAPα score ≥ 80) is an independent prognostic factor for lower metastasis-free survival (*p =* 0.027, HR = 2.867, 95% CI = 1.127–7.291) (Table 1). In contrast, this latter analysis failed to show a significant association of FAPα with clinicopathological factors for recurrence-free survival (*p =* 0.497) (Table 2) or overall survival (*p =* 0.979) (Table 3). These findings indicate that higher nuclear YAP1 and FAPα levels in stromal fibroblasts are associated with poorer prognosis of NPC patients.

**Table 1:** Univariate and Multivariate Analysis of YAP1/FAPα and Clinical Characteristics for Metastasis-Free Survival of NPC Patients

**YAP1**	Univariate analysis		Multivariate analysis	
*n* = 106	HR (95% CI)	*p* value	HR (95% CI)	*p* value
YAP1 Score (≥80 vs. < 80)	2.470 (1.057–5.776)	0.037	4.834 (1.857–12.579)	0.001
Treatment Days^a^	1.105 (1.008–1.21)	0.032	1.190 (1.069–1.325)	0.001
BED^a^	1.05 (0.875–1.155)	0.945	0.896 (0.772–1.040)	0.148
Age Group (≥48 vs. < 48)	0.830 (0.372–1.853)	0.65	0.727 (0.258–2.046)	0.546
Gender (Male vs. Female)	2.806 (0.837–9.410)	0.095	4.020 (1.007–16.059)	0.049
T-Stage (T_3/4_ vs. T_1/2_)	1.519 (0.675–3.419)	0.313	2.085 (0.750–5.800)	0.159
N-Stage (N_2/3_ vs. N_0/1_)	1.717 (0.762–3.867)	0.192	1.989 (0.817–4.843)	0.13
Chemotherapy (Yes vs. No)	0.891 (0.332–2.385)	0.818	0.616 (0.207–1.832)	0.384
Comorbidity (Yes vs. No)	1.161 (0.516–2.615)	0.718	1.416 (0.543–3.693)	0.477
Smoking (Yes vs. No)	1.516 (0.663–3.465)	0.324	1.098 (0.413–2.916)	0.851
Betel quid (Yes vs. No)	1.619 (0.671–3.905)	0.283	1.353 (0.382–4.798)	0.64
Alcohol (Yes vs. No)	1.263 (0.561–2.845)	0.572	0.913 (0.300–2.780)	0.873
**FAPα**	Univariate analysis		Multivariate analysis	
*n* = 143	HR (95% CI)	*p* value	HR (95% CI)	*p* value
FAPα Score (≥80 vs. < 80)	2.221 (0.995–4.959)	0.051	2.867 (1.127–7.291)	0.027
Treatment Days^a^	1.070 (0.994–1.152)	0.072	1.078 (0.984–1.180)	0.105
BED^a^	1.036 (0.952–1.127)	0.417	0.991 (0.911–1.078)	0.835
Age Group (≥48 vs. < 48)	0.742 (0.332–1.656)	0.466	0.526 (0.196–1.409)	0.201
Gender (Male vs. Female)	4.424 (1.040–18.813)	0.044	6.189 (1.334–28.724)	0.02
T-Stage (T_3/4_ vs. T_1/2_)	2.037 (0.871–4.762)	0.01	2.031 (0.748–5.512)	0.164
N-Stage (N_2/3_ vs. N_0/1_)	2.782 (1.153–6.710)	0.023	2.756 (1.111–6.833)	0.029
Chemotherapy (Yes vs. No)	0.730 (0.290–1.840)	0.505	0.594 (0.211–1.670)	0.323
Comorbidity (Yes vs. No)	1.392 (0.577–3.357)	0.462	1.047 (0.396–2.769)	0.926
Smoking (Yes vs. No)	1.114 (0.495–2.508)	0.794	0.482 (0.184–1.264)	0.138
Betel quid (Yes vs. No)	1.454 (0.603–3.508)	0.404	1.231 (0.403–3.762)	0.715
Alcohol (Yes vs. No)	1.181 (0.505–2.759)	0.701	0.928 (0.289–2.983)	0.9

^a^continuous variable; *BED*, Biologically effective dose, *HR* Hazard ratio, *CI* Confidence interval

**Table 2:** Univariate and Multivariate Analysis of YAP1/FAPα, and Clinical Characteristics for Recurrence-Free Survival of NPC Patients

**YAP1**	Univariate analysis		Multivariate analysis	
n = 106	HR (95% CI)	*p* value	HR (95% CI)	*p* value
YAP1 Score (≥80 vs. < 80)	2.255 (0.899–5.653)	0.083	3.841 (1.356–10.883)	0.011
Treatment Days^a^	1.099 (0.996–1.212)	0.06	1.203 (1.073–1.349)	0.002
BED^a^	0.943 (0.805–1.103)	0.46	0.884 (0.743–1.052)	0.164
Age Group (≥48 vs. < 48)	0.641 (0.262–1.568)	0.329	0.433 (0.133–1.416)	0.166
Gender (Male vs. Female)	1.642 (0.549–4.912)	0.375	1.631 (0.409–6.501)	0.488
T-Stage (T_3/4_ vs. T_1/2_)	2.043 (0.815–5.123)	0.128	5.354 (1.416–20.249)	0.013
N-Stage (N_2/3_ vs. N_0/1_)	1.541 (0.638–3.719)	0.336	1.388 (0.484–3.979)	0.541
Chemotherapy (Yes vs. No)	0.649 (0.249–1.690)	0.376	0.620 (0.208–1.849)	0.391
Comorbidity (Yes vs. No)	0.905 (0.361–2.269)	0.832	1.825 (0.579–5.752)	0.304
Smoking (Yes vs. No)	1.384 (0.566–3.386)	0.477	1.242 (0.384–4.020)	0.717
Betel quid (Yes vs. No)	2.361 (0.941–5.925)	0.067	0.769 (0.201–2.940)	0.702
Alcohol (Yes vs. No)	3.478 (1.386–8.727)	0.008	4.211 (1.217–14.574)	0.023
**FAPα**	Univariate analysis		Multivariate analysis	
n = 143	HR (95% CI)	*p* value	HR (95% CI)	*p* value
FAPα Score (≥80 vs. < 80)	1.259 (0.528–2.946)	0.595	1.415 (0.519–3.857)	0.497
Treatment Days^a^	1.124 (1.046–1.209)	0.002	1.170 (1.063–1.288)	0.001
BED^a^	1.046 (0.958–1.143)	0.313	1.060 (0.967–1.162)	0.216
Age Group (≥48 vs. < 48)	0.888 (0.385–2.049)	0.781	0.806 (0.293–2.219)	0.677
Gender (Male vs. Female)	1.311 (0.484–3.555)	0.594	0.749 (0.237–2.373)	0.624
T-Stage (T_3/4_ vs. T_1/2_)	2.209 (0.900–5.420)	0.084	2.339 (0.798–6.854)	0.121
N-Stage (N_2/3_ vs. N_0/1_)	2.428 (0.990–5.955)	0.053	2.176 (0.823–5.753)	0.117
Chemotherapy (Yes vs. No)	0.648 (0.253–1.656)	0.365	0.496 (0.164–1.500)	0.214
Comorbidity (Yes vs. No)	1.273 (0.519–3.123)	0.598	1.570 (0.569–4.332)	0.383
Smoking (Yes vs. No)	1.777 (0.725–4.359)	0.209	1.015 (0.330–3.125)	0.979
Betel quid (Yes vs. No)	2.135 (0.895–5.092)	0.087	0.947 (0.318–2.824)	0.922
Alcohol (Yes vs. No)	3.664 (1.565–8.577)	0.003	6.015 (1.839–19.669)	0.003

^a^continuous variable. *BED* Biologically effective dose, *HR* Hazard ratio, *CI* Confidence interval

**Table 3:** Univariate and Multivariate Analysis of YAP1/FAPα and Clinical Characteristics for Overall Survival of NPC Patients

**YAP1**	Univariate analysis		Multivariate analysis	
n = 106	HR (95% CI)	*p* value	HR (95% CI)	*p* value
YAP1 Score (≥80 vs. < 80)	1.447 (0.782–2.679)	0.239	2.402 (1.218–4.737)	0.011
Treatment Days^a^	1.074 (0.999–1.156)	0.053	1.142 (1.049–1.242)	0.002
BED^a^	0.982 (0.882–1.093)	0.737	0.887 (0.791–0.995)	0.04
Age Group (≥48 vs. < 48)	1.086 (0.587–2.009)	0.792	0.632 (0.288–1.389)	0.253
Gender (Male vs. Female)	2.412 (1.014–5.738)	0.046	3.657 (1.356–9.862)	0.01
T-Stage (T_3/4_ vs. T_1/2_)	1.956 (1.034–3.701)	0.039	3.584 (1.579–8.139)	0.002
N-Stage (N_2/3_ vs. N_0/1_)	1.394 (0.752–2.582)	0.291	1.554 (0.787–3.069)	0.204
Chemotherapy (Yes vs. No)	0.901 (0.449–1.911)	0.77	1.180 (0.515–2.706)	0.696
Comorbidity (Yes vs. No)	1.923 (1.041–3.551)	0.037	3.277 (1.493–7.190)	0.003
Smoking (Yes vs. No)	1.417 (0.756–2.658)	0.277	0.945 (0.453–1.971)	0.879
Betel quid (Yes vs. No)	1.707 (0.870–3.348)	0.12	0.980 (0.408–2.355)	0.964
Alcohol (Yes vs. No)	1.361 (0.733–2.527)	0.329	1.286 (0.588–2.812)	0.528
**FAPα**	Univariate analysis		Multivariate analysis	
n = 143	HR (95% CI)	*p* value	HR (95% CI)	*p* value
FAPα Score (≥80 vs. < 80)	1.248 (0.890–2.259)	0.463	1.009 (0.521–1.953)	0.979
Treatment Days^a^	1.044 (0.988–1.103)	0.129	1.039 (0.978–1.104)	0.212
BED^a^	1.007 (0.939–1.081)	0.837	0.965 (0.887–1.049)	0.403
Age Group (≥48 vs. < 48)	1.456 (0.804–2.635)	0.215	1.340 (0.691–2.596)	0.386
Gender (Male vs. Female)	1.338 (0.681–2.631)	0.398	1.364 (0.623–2.990)	0.437
T-Stage (T_3/4_ vs. T_1/2_)	2.610 (1.403–4.855)	0.002	3.324 (1.603–6.894)	0.001
N-Stage (N_2/3_ vs. N_0/1_)	1.313 (0.738–2.338)	0.354	1.257 (0.672–2.351)	0.474
Chemotherapy (Yes vs. No)	0.766 (0.401–1.463)	0.419	0.525 (0.243–1.133)	0.101
Comorbidity (Yes vs. No)	1.432 (0.797–2.570)	0.23	1.686 (0.872–3.263)	0.121
Smoking (Yes vs. No)	1.046 (0.587–1.864)	0.878	0.893 (0.437–1.824)	0.757
Betel quid (Yes vs. No)	1.555 (0.831–2.909)	0.167	1.837 (0.867–3.893)	0.112
Alcohol (Yes vs. No)	0.978 (0.523–1.831)	0.946	0.716 (0.327–1.568)	0.404

^a^continuous variable. *BED* Biologically effective dose, *HR* Hazard ratio, *CI* Confidence interval

EBV-positive NPC cells release exosomes that facilitate the establishment of a protumoral niche. However, whether these EBV product-containing exosomes participate in remodeling of the NPC tumor stroma in general or activation of fibroblasts in particular remains unclear. Accordingly, we tested the hypothesis that exosomes released from NPC cells cause fibroblast activation and, consequently, a protumoral microenvironment. Exosomes isolated from EBV^+^ NPC cells (HK1EBV) or Burkitt’s lymphoma cells (rAkata) were characterized using microbeads conjugated with anti-CD9 antibodies, followed by examination of exosome structures using transmission electron microscopy (TEM). TEM images of exosomes revealed a vesicle structure with a diameter ranging from 50 to 150 nm (Fig. 1I), confirming that the isolated pellets were exosome-rich fractions. To determine whether exosomes could be internalized by primary fibroblasts isolated from nasopharyngeal biopsies, we added prelabeled exosomes to the fibroblast culture and monitored cells using time-lapse microscopy. Intracellular exosome signals were visible within ~ 1 hour of addition and lasted for 48 hours (Supplementary Fig. S3). Western blot analyses further showed that LMP1, a crucial EBV-encoded oncoprotein, was present not only in EBV-positive cancer cells but also in exosomes secreted from these cells (Fig. 1J). We next examined the protumoral effects of HK1EBV cell-derived exosomes on fibroblasts using a three-arm mouse xenograft model in which NOD/SCID mice were subcutaneously injected in the thigh with HK1EBV cells, HK1EBV cells together with fibroblasts, or HK1EBV cells together with exosome-stimulated fibroblasts. IHC analyses showed the presence of active YAP1 in tumor cells and stromal fibroblasts and demonstrated FAPα immunoreactivity exclusively in stromal fibroblasts (Fig. 1K and Supplementary Fig. S4). Moreover, stromal fibroblasts of tumors coinjected with exosomes exhibited elevated levels of active YAP1 and FAPα, concomitant with an enhanced fibrotic response within tumor tissues. Furthermore, tumor progression was significantly increased in the group injected with HK1EBV cells together with exosome-stimulated fibroblasts compared with the other two groups (Fig. 1L and Supplementary Fig. S5). Additional analyses demonstrated a positive correlation between the expression of active YAP1 and FAPα (*p =* 0.0001, *r =* 0.7671) (Fig. 1M), FAPα and trichrome fibrotic intensity (*p* < 0.0001, *r =* 0.8597) (Fig. 1N), and active YAP1 and fibrotic intensity (*p =* 0.0015, *r =* 0.6753) (Fig. 1O), findings consistent with our clinical observations. Together, our data strongly suggest that HK1EBV cell-derived exosomes mediate fibroblast activation through YAP1 activation and promote tumor progression.

### Exosomes containing EBV products regulate the fibrotic response and inflammatory signaling in fibroblasts

**Fig. 2:**
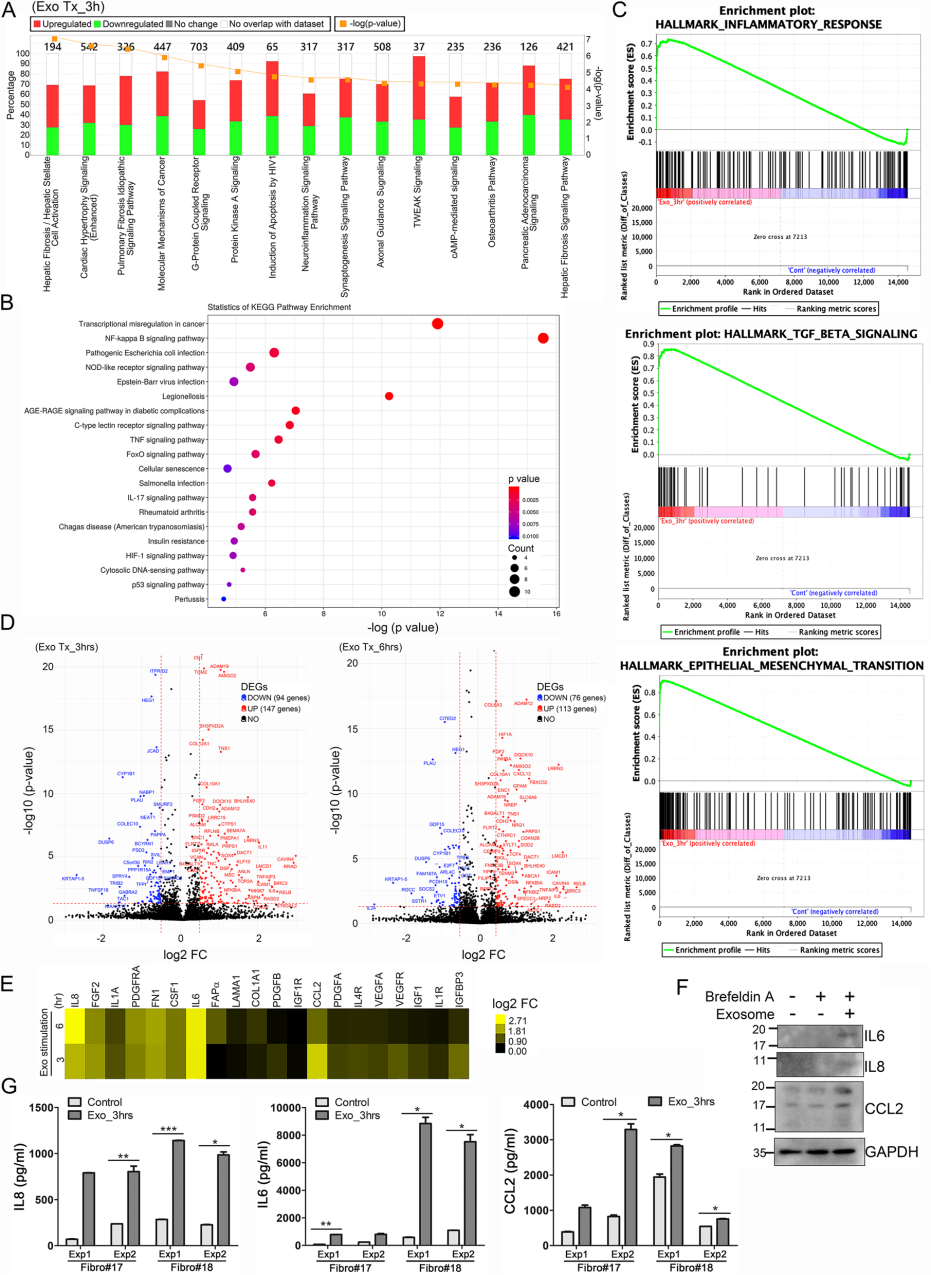
EBV product-containing exosomes enhance fibrotic and inflammatory signalings in fibroblasts. **A** Analysis of signaling pathways in fibroblasts stimulated with HK1EBV cell-derived exosomes compared with those exposed to medium. The top-scoring signaling pathways were identified using Ingenuity Pathway Analysis (IPA) software (QIAGEN). The identified pathways were ranked by significance [−log (*p*-value)]. The number of genes that met cutoff criteria [|log2(FC)| > 0.5 and *p* value < 0.05] in a given signaling pathway was shown. Total RNAs were harvested 3 hours post HK1EBV cell-derived exosome (10 μg/ml) stimulation. **B** Analysis of KEGG pathways in fibroblasts stimulated with HK1EBV cell-derived exosomes compared with those exposed to medium. **C** Gene set enrichment analysis (GSEA) in fibroblasts treated with HK1EBV cell-derived exosomes. GSEA plots for gene sets involved in inflammatory response, TGFβ signaling, and epithelial-mesenchymal transition were identified. Expression values were normalized to that of control fibroblasts. Significance threshold set at FDR < 0.25. **D** Volcano plots of transcriptomic analyses revealed differentially expressed genes (DEGs). Cutoff values were defined as |log2(FC)| > 0.5 and a *P*-value < 0.05. Total RNA was harvested 3 hours and 6 hours post exosome stimulation (10 μg/ml). **E** Expression heatmap of select genes upregulated by EBV product-containing exosomes. Expression levels were measured by qRT-PCR and normalized with respect to peptidylprolyl isomerase A (PPIA), used as an internal control. Values are expressed as log2 (fold change). **F** Western blot analysis of IL8, IL6, and CCL2 in fibroblasts. Protein lysates were harvested after 2-hour exosomal stimulation plus 4-hr treatment with Brefeldin A. GAPDH was used as a loading control. **G** Quantification of secreted IL8, IL6, and CCL2 in cell-free culture supernatants collected 3 hours post exosomal stimulation of fibroblasts. Data are presented as means ± SD of triplicate experiments (*p < 0.05**, *p* < 0.01, ***p < 0.001; paired t test)

To gain insight into the modulation of fibroblast biology by EBV-related exosomes, we performed next-generation RNA sequencing (RNA-seq) and measured differences in gene expression between the fibroblasts treated with HK1EBV-derived exosomes or culture medium (control). These analyses showed that several fibrosis-associated pathways and immune regulatory pathways were significantly enhanced in the fibroblasts treated with EBV product-containing exosomes (Fig. 2A and Supplementary Fig. S6). The results of a gene set enrichment analysis (GSEA) of KEGG signaling pathways showed that many EBV regulatory pathways, including the NF-κB, NOD-like receptor, TNF, FoxO, IL17 and hypoxia-inducible factor (HIF)-1 signaling pathways, were induced in these exosome-treated fibroblasts (Fig. 2B), suggesting that EBV-encoded products can exert effects via exosome transfer to noninfected cells. GSEA data further demonstrated that EBV-exosome stimulation enhanced the expression of genes involved in inflammatory responses, TGF-β signaling, and epithelial-mesenchymal transition (EMT) (Fig. 2C). Volcano plots of the RNA-seq results further identified biologically significant genes, including those associated with matrix remodeling, such as *FN1, IGFBP3, FGF2, TNC, COL6A3, COL10A1*, and *COL12A*1, and immune responses, such as *NF-κB1A, RELB, IL6, IL8, CCL2, CXCL12*, and *FAPα* (Fig. 2D). The expression levels of selected genes were further validated by qRT-PCR (Fig. 2E). Importantly, the transcriptomic data underscored the importance of cytokines, specifically revealing enhanced expression of IL8, IL6, and chemokine (C-C motif) ligand 2 **(**CCL2) in the fibroblasts treated with HK1EBV cell-derived exosomes. We therefore assessed the levels of the corresponding proteins in fibroblast lysates and culture supernatants and found that EBV exosomes markedly induced the production of IL6, IL8, and CCL2 (Fig. 2F and G, respectively).

### Viral product-containing exosomes enhance fibroblast activation and NPC cell growth

**Fig. 3:**
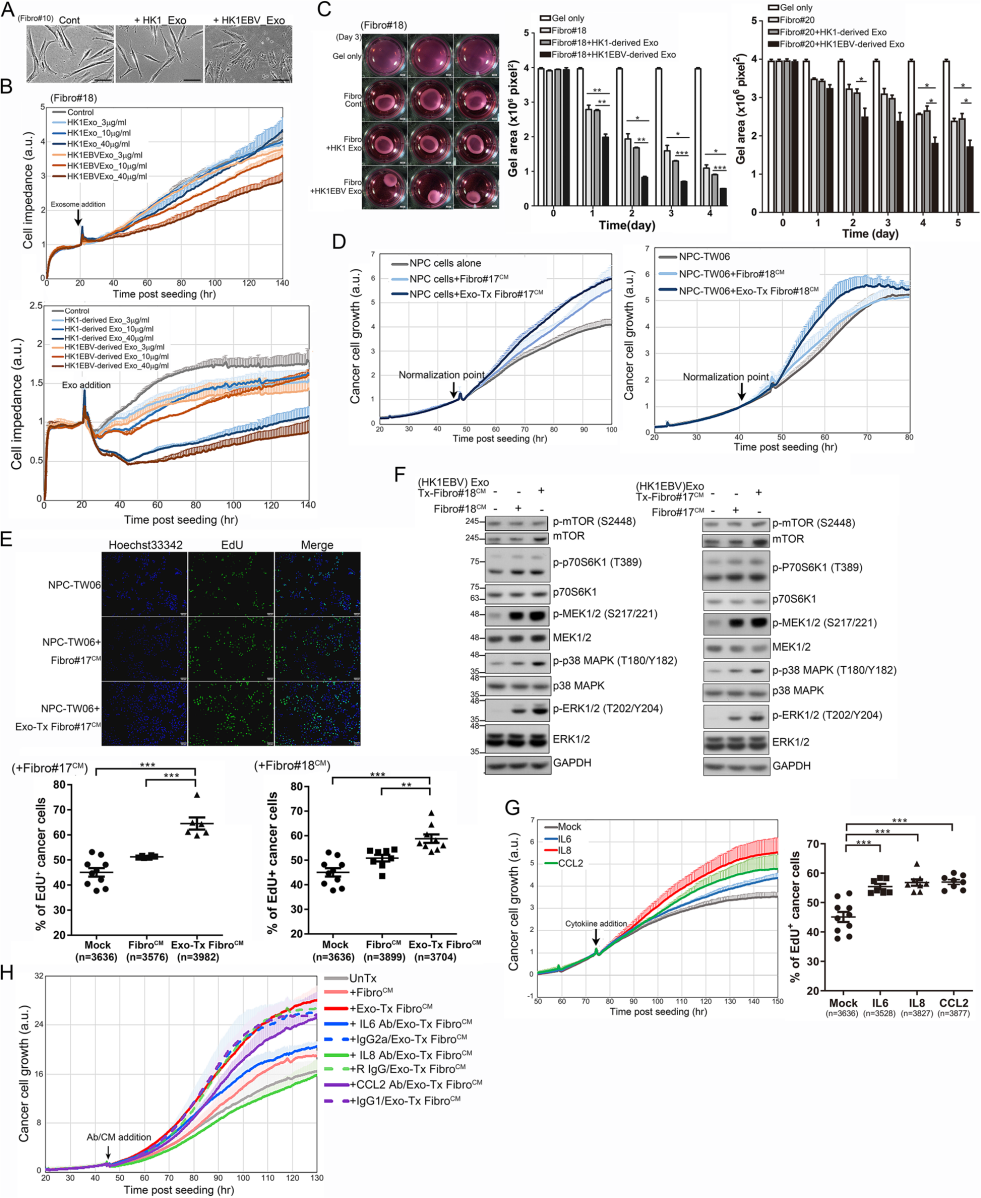
Exosomes containing EBV products promote fibroblast activation and NPC cell growth. **A** Phenotypic changes in fibroblasts. Primary fibroblasts were treated with PBS or exosomes (10 μg/ml) derived from HK1 cells or HK1EBV cells. Images were captured 24 hours post-exosome stimulation. Scale bar: 50 μm. **B** Real-time monitoring of changes in the fibroblasts stimulated with exosomes derived from HK1 cells, HK1EBV cells, or PBS (control). The black arrow indicates the time of exosome addition. Data are presented as the mean and SD of triplicate experiments. **C** Assessment of exosome-mediated effects on fibroblast contractility. Fibroblasts were treated with exosomes derived from HK1 or HK1EBV cells or medium only (control) for 3 days (left). Quantification of contractility was determined by measuring the gel area using ImageJ. Data are presented as the mean and SD (*p < 0.05, **p < 0.01, ***p < 0.001; paired t test). Scale bar, 2 mm. **D** Real-time proliferation assay of the NPC cells treated with cancer cell culture medium, culture supernatants harvested from the fibroblasts treated with HK1EBV cell-derived exosomes (Exo-Tx Fibro^CM^), or untreated fibroblasts (Fibro^CM^). Data are presented as the mean and SD of triplicate experiments. **E** Analysis of EdU incorporation in the NPC-TW06 cells treated with supernatants harvested from the fibroblasts treated with HK1EBV cell-derived exosomes or control medium for 10 hours, followed by EdU labeling (green) for 4 hours. Scale bar, 100 μm. EdU^+^ cells were quantified using CellSens software. (**p < 0.01; ***p < 0.001; Bonferroni’s multiple comparison test). **F** Analysis of pro-proliferative signaling pathways in NPC-TW06 cells. Protein lysates were harvested 30 minutes after exosomal treatment. GAPDH was used as a loading control. **G** Stimulation with IL6 (20 ng/ml), IL8 (50 ng/ml), or CCL2 (100 ng/ml) enhances NPC cell proliferation (right) and DNA synthesis (left)**.** EdU^+^ cells were quantified using CellSens software (Olympus) (**p < 0.01; ***p < 0.001; Bonferroni’s multiple comparison test). **H**. Depletion of IL6, IL8, or CCL2 by the addition of cytokine-specific neutralizing antibodies suppresses Exo-Tx Fibro^CM^-mediated NPC cell proliferation. NPC cells were pretreated with antibodies for one hour and then stimulated with conditioned medium. Data are presented as the mean and SD of triplicate experiments

We next investigated whether NPC-derived exosomes enhance the formation of CAFs, corresponding to local fibroblasts that have undergone activation by factors present in the TME. We found that EBV product-containing exosomes enhanced the formation of stress fibers and induced a smaller, myofibroblast-like phenotype, unlike that observed in the phosphate-buffered saline (PBS)- or HK1 cell exosome-treated groups (Fig. 3A and Supplementary Movies 1, 2, 3). To further evaluate exosome-mediated phenotypic changes in fibroblasts, we measured cell impedance. This analysis demonstrated that treatment of fibroblasts with HK1EBV cell-derived exosomes caused a concentration-dependent decrease in impedance compared to treatment with EBV-negative HK1 cell-derived exosomes (Fig. 3B). Since the population doubling time of primary fibroblasts ranged from 3.5 days to 5 days depending on cell passage number, alterations in cell impedance primarily reflect changes in cell shape and attachment, which are associated with stress fiber formation and mechanical transduction. We therefore assessed fibroblast contractility and found that the fibroblasts treated with HK1EBV cell-derived exosomes exhibited markedly increased contractile ability compared to the fibroblasts treated with PBS or HK1 cell-derived exosomes (Fig. 3C, left). A quantitative analysis revealed that EBV product-containing exosomes enhanced the contractile properties of fibroblasts, an effect that increased with the duration of stimulation (Fig. 3C, middle and right). Acquisition of a fibroblastic phenotype could indicate enhanced tumor progression. To confirm this possibility, we further assessed the impacts of exosome-stimulated fibroblasts on cancer cells. The results of these analyses showed that NPC cells cultured in conditioned medium (Exo-Tx Fibro^CM^) from the fibroblasts treated with HK1EBV cell-derived exosomes grew faster than those cultured in conditioned medium (Fibro^CM^) from untreated fibroblasts or cancer cell culture medium (mock-treated control) (Fig. 3D). This enhanced effect on NPC cell proliferation was further confirmed by an analysis of DNA synthesis using EdU incorporation assays (Fig. 3E, upper). Quantitative analyses of these assays showed that supernatants derived from the fibroblasts treated with HK1EBV cell-derived exosomes significantly enhanced DNA synthesis (Fig. 3E, lower) and activation of mTOR/p70S6K1, MEK1/2, ERK1/2, and p38 MAPK signaling (Fig. 3F), indicating enhanced cancer cell proliferation. To confirm the identity of factors involved in this cancer growth-promoting effect, we added selected cytokines to the culture medium and evaluated NPC cell proliferation. This analysis showed that stimulation with IL6, IL8, or CCL2 substantially enhanced NPC cell proliferation (Fig. 3G, left) and increased DNA synthesis (Fig. 3G, right). In contrast, depletion of IL6, IL8, or CCL2 by adding specific neutralizing antibodies to NPC cultures eliminated the cancer growth-promoting effect of Exo-Tx Fibro^CM^, while treatment with isotype IgG control antibodies did not affect this effect (Fig. 3H). Collectively, these data indicate that EBV exosomes not only enhance fibrotic responses but also regulate inflammatory signaling, which together alter the TME and facilitate NPC growth.

### YAP1 and FAPα are associated with the activation of fibroblasts by HK1EBV cell-derived exosomes

**Fig. 4:**
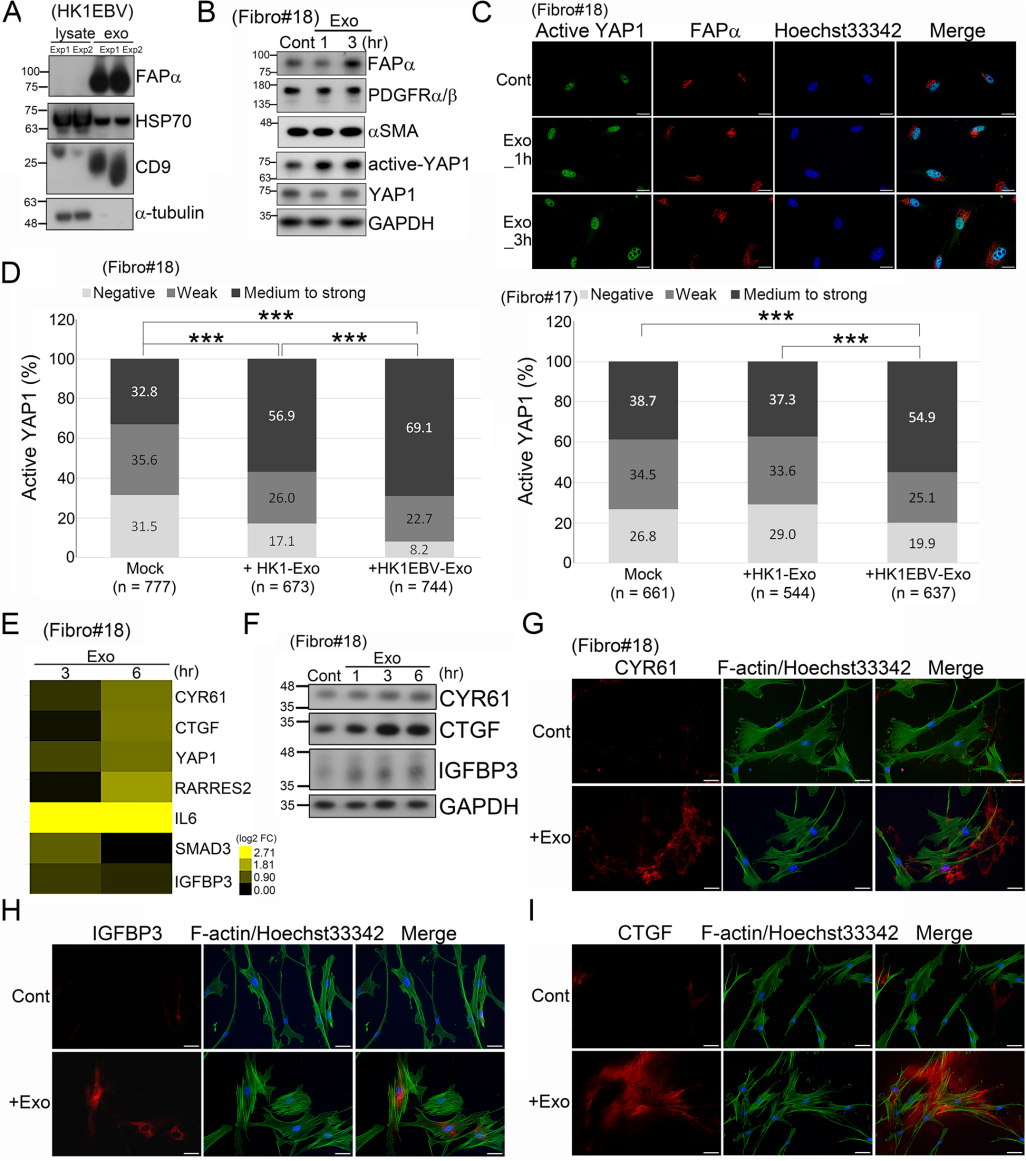
NPC-derived exosomes enhance activation of YAP1 signaling in fibroblasts. **A** Exosomes released from HK1EBV cells contain FAPα protein. **B** Treatment of fibroblasts with HK1EBV cell-derived exosomes (10 μg/ml) increased levels of FAPα and active YAP1. Total protein lysates were harvested 1 and 3 hours post exosome treatment. GAPDH was used a protein loading control. **C** Immunostaining for active YAP1 (green) and FAPα (red) in fibroblasts treated with exosomes derived from HK1EBV cells. Cells were fixed 1 or 3 hours post exosome stimulation. Nuclei were stained with Hoechst 33342. Scale bar, 20 μm. **D** Quantification of active YAP1 expression in fibroblasts treated with exosomes compared with that in untreated cells. Cells were fixed 3 hours post exosome stimulation. Differential expression of active YAP1 was defined based on fluorescence intensities. Cell counts were determined using ImageJ software (***p < 0.001; chi-square test). **E** Assessment of mRNA expression of YAP1 downstream target genes. Total RNAs were extracted 3 or 6 hours after stimulating with HK1EBV-derived exoosmes. GAPDH was used as an internal control. Expression values were normalized to that of untreated fibroblasts. Values were expressed as mean and SD of two independent experiments. **F** Western blot analysis of YAP1 downstream proteins, CYR61, CTGF, and IGFBP3. Fibroblast protein lysates were harvested 1, 3, and 6 hours post exosome treatment. GAPDH was used as a loading control. **G–I** Immunofluorescence staining for YAP1 downstream molecules CYR61 (**G**), CTGF (**H**), and IGFBP3 (**I**) in fibroblasts treated with exosomes derived from HK1EBV cells. Cells were fixed 3 hours post exosome stimulation. Alexa Fluor 488 phalloidin (green) was used to stain F-actin. Nuclei were stained with Hoechst 33342. Scale bars, 50 μm

Recently, YAP1 was identified as a key regulator of CAF activation [42, 44]. We investigated the potential mechanism of YAP1 by which EBV product-containing exosomes dysregulate fibroblast function within the tumor microenvironment. In addition to EBV-LMP1, we first found that exosomes released from HK1EBV cells contained abundant FAPα protein (Fig. 4A), which may be subsequently taken up by surrounding cells or deposited in the extracellular matrix (ECM). Fibroblasts treated with these viral product-containing exosomes exhibited enhanced levels of active YAP1 and FAPα (Fig. 4B and C), but the levels of PDGFRα/β and α-SMA were not affected (Fig. 4B). We further stratified active YAP1 into three levels—negative, weak and medium-to-strong—based on expression intensity and quantified the positivity of each group. This analysis showed that the percentage of cells strongly expressing active YAP1 was higher in the fibroblasts treated with HK1EBV cell-derived exosomes than in the untreated fibroblasts or those treated with exosomes from HK1 cells (Fig. 4D). These EBV product-containing exosomes also enhanced the expression of YAP1 downstream targets, including cysteine rich angiogenic inducer 61 (CYR61), connective tissue growth factor (CTGF), and insulin-like growth factor-binding protein 3 (IGFBP3) (Fig. 4E and F), indicating that HK1EBV cell-derived exosomes are capable of potentiating YAP1 activity. In addition, CYR61, CTGF, and IGFBP3 showed widely distributed, enhanced expression in the cytosol and ECM of the fibroblasts treated with these exosomes (Fig. 4G–I). Taken together, these findings suggest that EBV product-containing exosomes convert fibroblasts into CAFs by enhancing YAP1 signaling and increasing FAPα levels, with subsequent expression and secretion of CYR61, CTGF, and IGFBP3 likely contributing to reconstruction of the NPC microenvironment.

### Suppression of YAP1 abolishes functions mediated by HK1EBV cell-derived exosomes associated with fibroblast activation

**Fig. 5:**
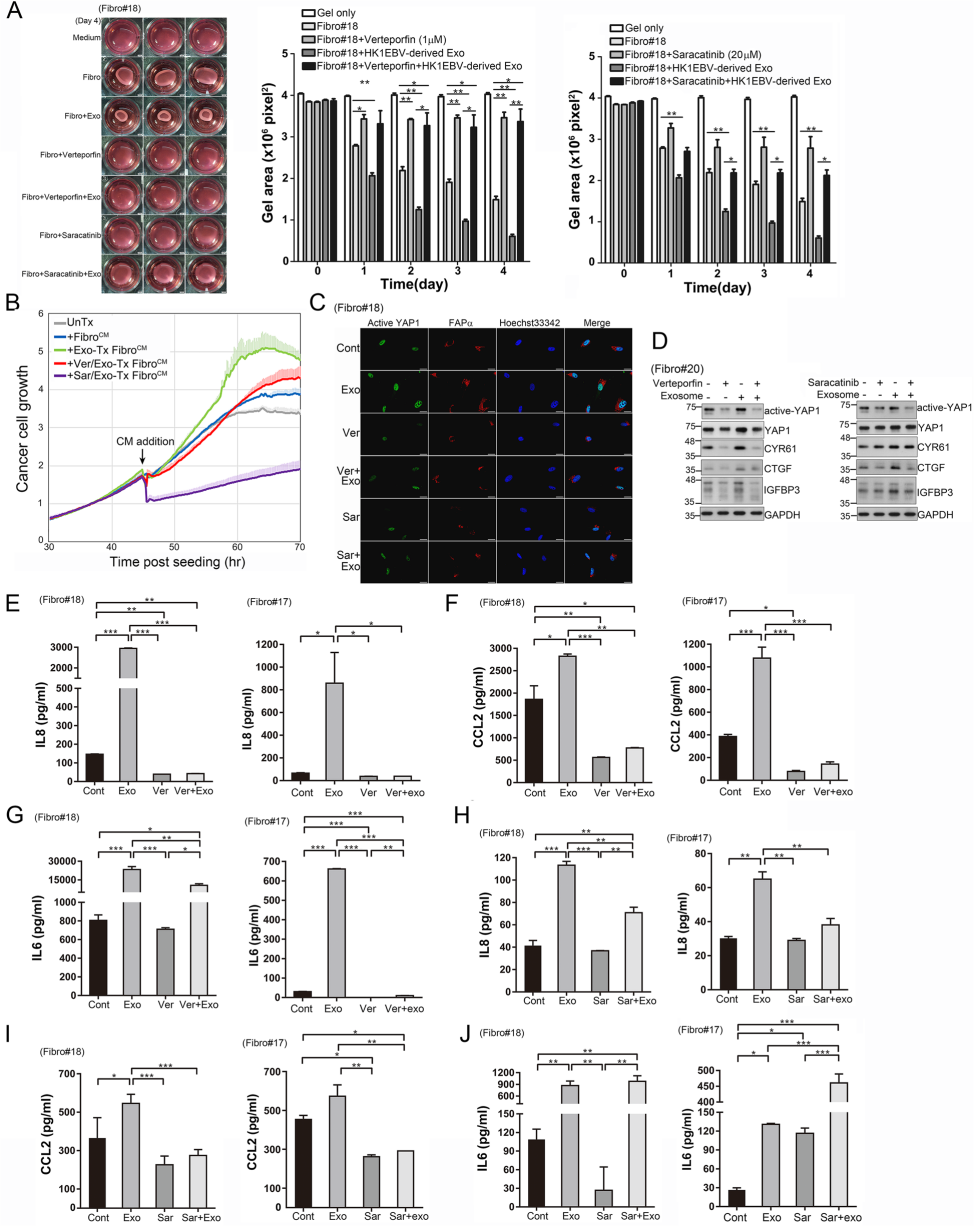
Inhibition of YAP1 prevents EBV exosome-induced fibroblast activation. **A** Assessment of verteporfin- and saracatinib-mediated inhibition of HK1EBV exosome-induced fibroblast contractility. **B** YAP1 inhibition in fibroblasts decreases EBV exosome-promoted NPC cell proliferation. The culture medium of the fibroblasts treated with inhibitor or vehicle was replaced with fresh medium containing 1% FBS after 5 hours of drug treatment, followed by 3 hours of exosomal stimulation. Fibroblast supernatants were then collected and used to treat NPC cells. Data are presented as the mean and SD of triplicate experiments. **C** Immunofluorescence staining of active YAP1 (green) and FAPα (red) in the fibroblasts treated with HK1EBV cell-derived exosomes, alone or together with verteporfin or saracatinib. Cells were fixed 3 hours after exosome stimulation. Blue, nuclear staining. Scale bars, 20 μm. **D** Western blot analysis of active YAP and its downstream target proteins in the fibroblasts treated with HK1EBV-derived exosomes, alone or together with verteporfin (3 μM) or saracatinib. Protein lysates were harvested 3 hours after exosome treatment. GAPDH was used as a loading control. **E–G** Assessment of verteporfin-mediated suppression of the levels of IL8 (**E**), CCL2 (**F**), and IL6 (**G**) released from the fibroblasts treated under the indicated conditions. **H–J,** Assessment of saracatinib-mediated suppression of the production of IL8 (**H**), CCL2 (**I**), and IL6 (**J**) released from fibroblasts. Unless otherwise indicated, verteporfin (1 μM) or saracatinib (20 μM) was added to the growth medium of fibroblasts 2 hours prior to exosome (10 μg/ml) treatment. Data are presented as the mean and SD of triplicate experiments (*p < 0.05, **p < 0.01, ***p < 0.001; Bonferroni’s multiple comparison test)

Having demonstrated that YAP1 is important in regulating fibroblast activation through the uptake of HK1EBV cell-derived exosomes, we next investigated whether inhibiting YAP1 activity reversed the protumoural characteristics described above. To this end, we used verteporfin, which blocks the association of YAP with TEAD and thereby disrupts YAP-related transcriptional activity, and saracatinib, which is an SRC kinase inhibitor that was recently reported to exert suppressive activities against YAP1 in NPC [38, 45]. We first determined the cytotoxic effects of these two YAP1 inhibitors, quantified as half-maximal inhibitory concentration (IC_50_) values, on two primary fibroblast strains (Fibro#17 and #18) using a real-time cell-impedance analyzer. The IC_50_ values of verteporfin and saracatinib for Fibro#17 were 2.58 and 20.5 μM, respectively (Supplementary Fig. S7A and B), and the corresponding IC_50_ values for Fibro#18 were 6.11 and 20.03 μM (Supplementary Fig. S7C and D). Optimal concentrations of verteporfin (1–3 μM) and saracatinib (20 μM) were used in subsequent functional assays. As shown in Fig. 5A, verteporfin or saracatinib treatment inhibited HK1EBV exosome–induced cell contractility (Fig. 5A). Furthermore, administration of YAP1 inhibitors in fibroblasts substantially suppressed Exo-Tx Fibro^CM^-mediated NPC cell proliferation (Fig. 5B). Immunofluorescence analyses showed decreased expression of active YAP1 and FAPα in the inhibitor–treated fibroblasts compared with the EBV-exosome–stimulated cells (Fig. 5C). Importantly, verteporfin or saracatinib treatment also suppressed exosome-mediated activation of YAP1 and its downstream molecules CYR61, CTGF, and IGFBP3 in fibroblasts (Fig. 5D). Likewise, the results of enzyme-linked immunosorbent assays (ELISAs) showed that the amounts of IL-8, CCL2, and IL6 secreted into the culture medium of fibroblasts were decreased by treating cells with verteporfin (Fig. 5E–G). Treatment of fibroblasts with saracatinib also suppressed EBV exosome-induced production of IL8 and CCL2 (Fig. 5H and I) but not IL6 (Fig. 5J). This discrepancy between the effects of verteporfin and saracatinib on IL6 production could be because saracatinib acts on multiple targets. Collectively, our findings demonstrate that YAP1 activation is the key event leading to fibroblast activation and that pharmacological inhibition of YAP1 prevents fibroblast activation induced by HK1EBV cell-derived exosomes.

### Stromal YAP1 and FAPα expression are negatively correlated with the abundance of GZMB^+^ CD8 T cells

**Fig. 6:**
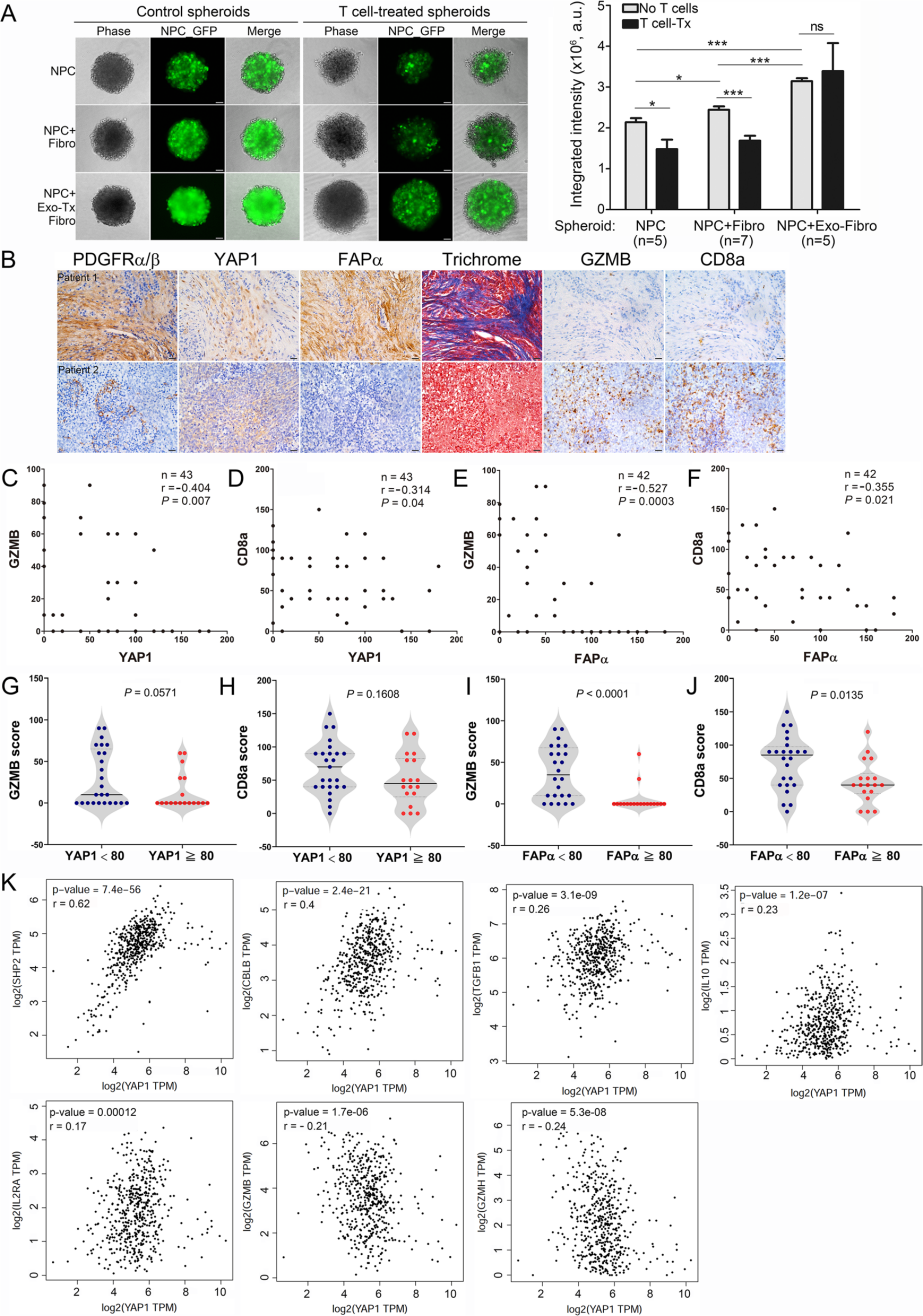
Correlation analyses of YAP1, FAPα, GZMB, and CD8a expression in human tumor biopsies. **A** T cell-mediated cytotoxicity on 3D NPC spheroids. PBMC-derived T cells were activated by stimulation with human rIL2 and Dynabeads magnetic beads coated with anti-CD3 and anti-CD28 antibodies. Activated T cells were added to 1-day NPC-TW06-GFP spheroids with an effect/target ratio of 3:1. Images were captured after 24 hours of T cell addition. The integrated GFP intensity of spheroids was calculated using ImageJ software (*p < 0.05, ***p < 0.001; Welch’s t test). **B** Representative IHC images of YAP1, FAPα, fibrotic intensity, GZMB, and CD8a in consecutive NPC tissue sections. Scale bar: 50 μm. **C–F** Correlation analyses based on IHC scores (Spearman’s correlation test). The correlations shown include those between YAP1 and GZMB (**C**), YAP1 and CD8a (**D**), FAPα and GZMB (**E**), and FAPα and CD8a (**F**). **G–J** Different levels of GZMB or CD8a in NPC tumors. Tumor samples were classified into two groups based on their YAP1 or FAPα score (score < 80 vs. score ≥ 80) detected in fibroblasts of tumor samples. GZMB score according to YAP1 expression (**G**). CD8a score according to YAP1 expression **(H)**. GZMB score according to FAPα expression **(I)**. CD8a score according to FAPα expression **(J)**. *p* values were calculated using the Mann-Whitney U test. **K** Correlation analysis between the expression of YAP1 and immune regulatory molecules. Expression data were extracted from the TCGA head and neck squamous cell carcinoma RNA sequencing dataset. Spearman’s correlation test was used to calculate the correlation between the mRNA expression levels of two select genes

Our data support the hypothesis that stimulation with NPC-derived exosomes activates CAFs, causing them to acquire protumorigenic properties. In addition, multiple inflammatory pathways and the expression of immune-suppressive molecules, such as IL8, IL6, CCL2, FGF2 and CSF1, were enhanced in the EBV-exosome–stimulated fibroblasts. Thus, these activated fibroblasts may also significantly impact tumor immunity. Cytotoxic T lymphocytes—the major adaptive immune cells in the TME—are vital for anticancer immunity. Thus, we established a 3D tumor spheroid model to recapitulate features of the TME, in which immune cells need to infiltrate a 3D structure to attack the target cells. After 24 hours of coculture, the control NPC-GFP cells formed loosely aggregated multicellular spheroids, and the NPC + Fibro spheroids showed a slight increase in both size and GFP intensity, but the NPC + Exo-Tx Fibro spheroids formed tight spheroids with strong GFP intensity (Fig. 6A, left). Twenty-four hours post-T cell addition, lysis of target cells was detected in both the NPC and NPC + Fibro spheroids compared to their untreated controls, while target cell cytotoxicity was minimally detected in the NPC + Exo-Tx Fibro spheroids. Quantification results displayed significant T cell-induced cytotoxicity on the NPC and NPC + Fibro spheroids. However, the NPC + Exo-Tx Fibro spheroids were resistant to T cell attack (Fig. 6A, right). These data support the role of EBV-exosomes in T cell immune regulation. We next assessed the expression of the cytotoxic enzyme granzyme B (GZMB) and CD8a in human NPC tumors. IHC analyses revealed enriched GZMB^+^ CD8 T cell infiltration in tumors harboring stromal fibroblasts with lower YAP1 and FAPα immunoreactivities and diminished GZMB^+^ CD8 T cell infiltration in tumors whose resident stromal fibroblasts exhibited higher YAP1 and FAPα immunoreactivities (Fig. 6B). Additional analyses revealed inverse correlations between YAP1 and GZMB (*p =* 0.007, *r =* − 0.404) (Fig. 6C), YAP1 and CD8a (*p =* 0.04, *r =* − 0.314) (Fig. 6D), FAPα and GZMB (*p =* 0.0003, *r =* − 0.527) (Fig. 6E), and FAPα and CD8a (*p =* 0.021, *r =* − 0.355) (Fig. 6F). Moreover, GZMB and CD8a immunoreactivities were enhanced in tumors harboring stromal fibroblasts expressing lower levels of YAP1 and FAPα, and were decreased in tumors in which stromal fibroblasts expressed higher levels of YAP1 and FAPα (Fig. 6G–J). To provide more supporting evidence that YAP1 and FAPα induce an immunosuppressive T cell phenotype, we interrogated the Cancer Genome Atlas (TCGA) database for correlations between YAP1/FAPα expression and immune regulatory molecules in head-and-neck squamous cell carcinoma. Importantly, we found that the levels of YAP1 were positively correlated with the expression of the immune regulatory molecules SHP2 (*p =* 7.4 × 10^− 56^, *r =* 0.62), CBLB (*p =* 2.4 × 10^− 21^, *r =* 0.4), TGFβ, IL10, and IL2RA but were inversely correlated with the expression of the cytotoxic enzymes GZMB and GZMH (Fig. 6K). Similar results were found for the correlation between the expression of FAPα and that of the above immune regulatory genes (Supplementary Fig. S8). These findings imply that fibroblasts activated by NPC-derived exosomes create an immunosuppressive microenvironment by inducing a loss of function in cytotoxic T lymphocytes. Together, these results suggest that the abundance of cytotoxic CD8 T cells is inversely related to the activity of YAP1 and FAPα in stromal fibroblasts and that these interactions between EBV-positive NPC tumors and stroma drive immune suppression.

**Fig. 7:**
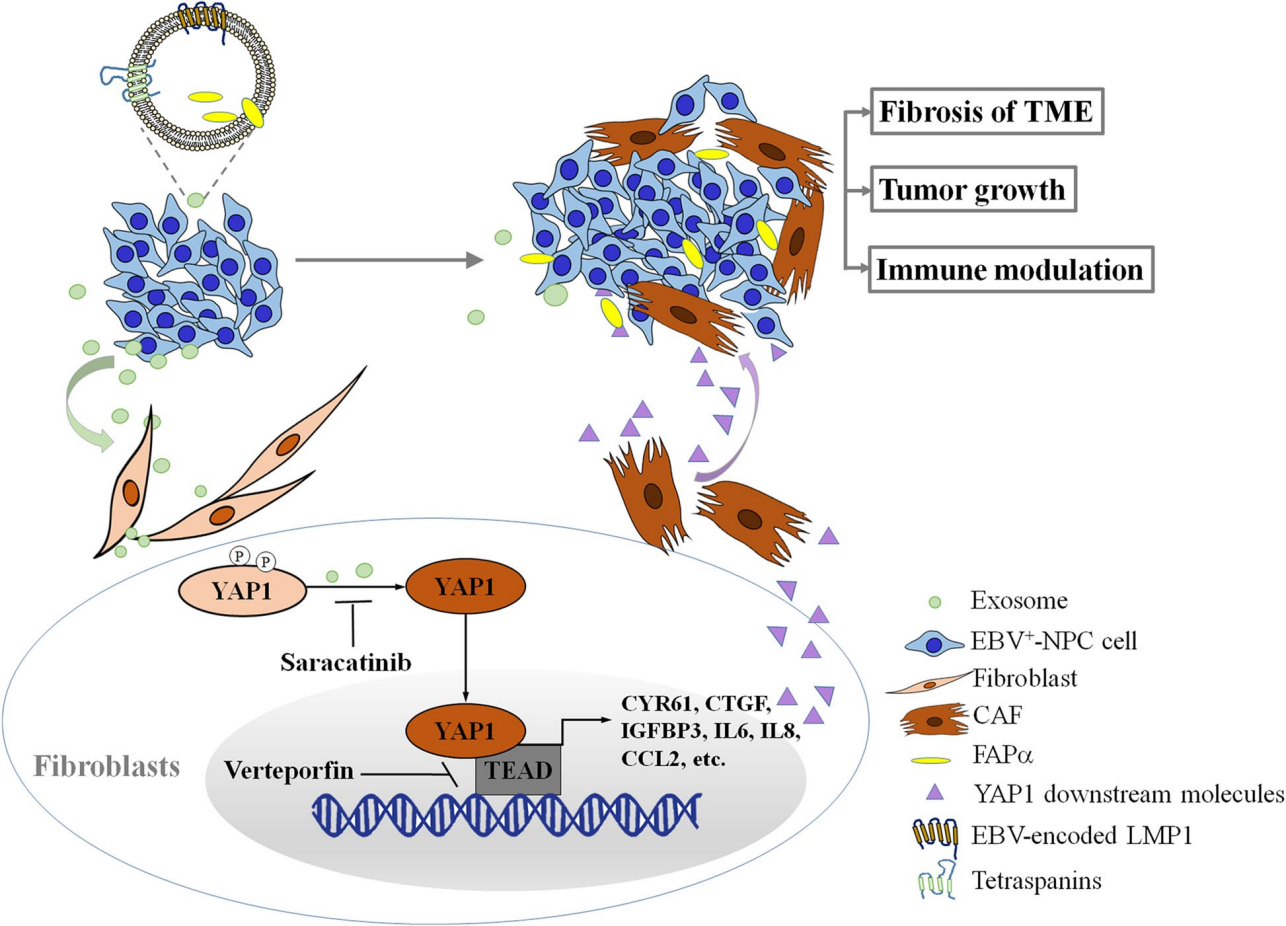
Model for activation of fibroblasts by EBV product-containing exosomes and biological effects in the NPC tumor microenvironment

On the basis of our findings, we propose a model for the effects of NPC-derived exosomes on stromal fibroblasts of NPC tumors. In this model, exosomes increase FAPα levels and activate YAP1 signaling to promote CAF-mediated effects, including fibrosis and tumor growth, while concurrently inducing an immunosuppressive TME that benefits NPC progression (Fig. 7).

## Discussion

EBV-positive NPC cells are known to modulate the functions of neighboring cells through the release of tumor-promoting factors into the microenvironment and the transfer of EBV-encoded products, such as miRNAs and LMP1, to EBV-negative cancer cells and immune cells through exosome cargos. In the present study, we demonstrated that NPC-derived EBV product-containing exosomes induce the activation of fibroblasts through enhanced FAPα release and YAP1 signaling. Internalization of these exosome cargos by fibroblasts contributed to enhanced tumor growth and fibrotic responses in mouse xenografts, strongly implicating the protumoral and profibrotic roles of EBV-related exosomes in the TME in disease-related deterioration. Moreover, higher levels of YAP1 and FAPα in stromal fibroblasts were both correlated with NPC metastasis. FAPα is highly expressed in CAFs and is known to promote a protumoral TME through remodeling of the tumor stroma [46]. Intriguingly, NPC-derived exosomes encapsulated high levels of FAPα, whereas FAPα was nearly absent in the cell itself. One reasonable explanation for this finding is that cancer cells eliminate proteins or mRNAs that are not of endogenous origin via exosomal cargo and simultaneously remodel the TME to benefit themselves. However, YAP1 is closely regulated by the extracellular environment, including ECM factors, and through cell-cell adhesion and mechanotransduction [47]. FAPα is a serine protease that can cleave substrates in the ECM; such cleavage leads to tissue remodeling and thereby further enhances YAP1 activity. The activation of YAP1 in fibroblasts enhances the release of CYR61, CTGF, and IGFBP3 into ECM. Therefore, it is likely that these factors create a positive feedback loop that reinforces CAF activation and reconstruction of NPC microenvironment. We further showed that selective YAP inhibition in fibroblasts attenuated exosome-induced, YAP1-regulated fibroblast activation, suggesting that YAP1 inhibition might be a potential approach for attenuating tumor progression.

EBV-infected NPC, characterized by heavy immune cell infiltration, highlights the complicated relationships among tumor cells, immune cells, and EBV [48]. By limiting the number of expressed viral proteins, EBV can escape host immune surveillance [11]. Furthermore, the expression of EBV-encoded products, such as LMP1, vIL-10 and miRNAs, permits immune evasion, allowing EBV to escape immune surveillance during latency and productive infection [12]. Our data showed that EBV product-containing exosomes significantly increased the expression of genes involved in multiple inflammatory pathways and enhanced the release of immune suppressive cytokines, such as CCL2, IL8 and IL6, from fibroblasts. In a 3D coculture model, fibroblasts treated with these EBV-exosomes promoted NPC resistance to T cell attack. Furthermore, a lower density of YAP1 and FAPα is associated with an increased density of tumor-infiltrating CD8a^+^ T lymphocytes with a cytotoxic immune signature, implying that fibroblasts may also engage in recruiting regulatory leukocytes to the TME. These factors released by exosome-activated fibroblasts impact immune cells and tumor cells, creating a positive feedback loop that reinforces cancer progression.

## Conclusions

Collectively, our findings provide strong evidence linking YAP1 and FAPα in NPC stromal activation and metastasis and further suggest that the levels of YAP1 and FAPα may serve as independent predictors of NPC metastasis. Additionally, our observations reveal a molecular basis for the intercellular communication between EBV-positive NPC cells and the TME, with particular emphasis on the transmission of viral product-containing exosomes to primary fibroblasts, demonstrating that viral product-containing exosomes enhance the release of FAPα, activation of YAP1 signaling, fibrotic response, immune suppression, and tumor growth in NPC. Understanding the biological roles of EBV product-containing exosomes in the TME should aid in the development of strategies for breaking down the “soil” for tumor progression.

## Supplementary Information

**Additional file 1: Supplementary Table S1.** Baseline Characteristics of the YAP1 Study Population.

**Additional file 2: Supplementary Table S2.** Baseline Characteristics of the FAPα Study Population.

**Additional file 3: Supplementary Table S3.** Primer information for quantitative RT-PCR analysis.

**Additional file 4: Supplementary Fig. S1.** Uncropped western blots for figures.

**Additional file 5: Supplementary Fig. S2.** Representative IHC images of PDGFRα/β, YAP1, FAPα, and trichrome staining in paraffin-embedded consecutive human NPC tissue sections. Black arrows signify fibroblast-like cellular structures. Yellow marked zones indicate selected tumor beds. Fibrosis (blue) within tissue sections was evaluated using trichrome staining. Scale bar, 20 μm.

**Additional file 6: Supplementary Fig. S3**. Uptake of exosomes by primary fibroblasts. Exosomes were pre-labeled with green fluorescent dye and added to cultures of fibroblasts. Images were captured 24 hours post exosome stimulation. Scale bar, 50 μm.

**Additional file 7: Supplementary Fig. S4**. Representative IHC images of PDGFRα/β, active YAP1, FAPα, and trichrome staining in paraffin-embedded consecutive NPC xenografts. Black arrows signify fibroblast-like cellular structures. Yellow marked zones indicate selected tumor beds. Fibrosis (blue) within tumor sections was evaluated using trichrome staining. Scale bar, 20 μm.

**Additional file 8: Supplementary Fig. S5.** Images of xenografts dissected from NOD/SCID mice after 7-week subcutaneous injection. HK1EBV cells, HK1EBV cells together with fibroblasts, or HK1EBV cells together with exosome-treated fibroblasts were subcutaneously injected into the legs of mice (*n* = 8–9 mice/group).

**Additional file 9: Supplementary Fig. S6.** Deregulated signaling pathways in fibroblasts treated with EBV product-containing exosomes. Analysis of signaling pathways in fibroblasts stimulated with HK1EBV cell-derived exosomes compared with those exposed to medium. The top-scoring signaling pathways were identified using Ingenuity Pathway Analysis (IPA) software (QIAGEN). The identified pathways were ranked by significance [−log (*p*-value)]. The number of genes that met cutoff criteria [|log2(FC)| > 0.5 and *p* value < 0.05] in a given signaling pathway was shown. Total RNAs were harvested 6 hours post HK1EBV cell-derived exosome (10 μg/ml) stimulation.

**Additional file 10: Supplementary Fig. S7.** Cytotoxicity assessments of different doses of verteporfin or saracatinib in primary fibroblasts. **A–B** Real-time measurement of cell survival in response to various doses of verteporfin (**A**) or saracatinib (**B)** treatment in Fibro#17 cells. **C–D** Measurement of cell survival in response to various doses of verteporfin (**C**) or saracatinib (**D**) treatment in Fibro#18 cells. Data are presented as means and SD of **quadruplicate** experiments (left panels). The drug-induced cell toxicity was determined by calculating the IC_50_ after treatment (right panels). Normalized cell growth was displayed against the logarithm of concentration. The values of IC_50_ were calculated using the RTCA software (ACEA Biosciences).

**Additional file 11: Supplementary Fig. S8.** Correlation analysis between expression of FAPα and immune regulatory molecules. Expression data were extracted from the *TCGA head and neck squamous cell carcinoma RNA sequencing dataset.* Spearman’s correlation test was used to calculate the correlation between mRNA expressions of two select genes.

**Additional file 12: Supplementary movie S1.** Time-lapse live cell imaging of primary fibroblasts stimulated with vehicle (PBS).

**Additional file 13: Supplementary movie S2.** Time-lapse live cell imaging of primary fibroblasts stimulated with exosomes derived from HK1 cells.

**Additional file 14: Supplementary movie S3.** Time-lapse live cell imaging of primary fibroblasts stimulated with exosomes derived from HK1EBV cells.

## Data Availability

The datasets supporting the conclusions of this article are included within the article and its supplementary data files. The RNA sequencing data has been deposited in the Gene Expression Omnibus (GSE197560*)* database and will be released upon publication. The online version of Supplementary data will be available at Journal’s official website upon publication.

## Abbreviations

NPC: Nasopharyngeal carcinoma
EBV: Epstein-Barr virus
YAP1: Yes-associated protein 1
FAPα: Fibroblast activation protein alpha
IHC: Immunohistochemistry
CAF: Cancer-associated fibroblast
IL8: Interleukin 8
CCL2: C-C motif chemokine ligand 2
IL6: Interleukin 6
α-SMA: Smooth muscle actin alpha
FSP-1: Fibroblast specific protein 1
VEGF: Vascular endothelial growth factor
CXCL12: C-X-C motif chemokine ligand 12
TME: Tumor microenvironment
PDGF: Platelet-derived growth factor
LMP1: EBV-encoded latent membrane protein 1
NF-κB: Nuclear factor kappa-light-chain-enhancer of activated B cells
STAT3: Signal transducer and activator of transcription 3
IL1: Interleukin 1
CXCL1: C-X-C motif chemokine ligand 1
LIF: Leukemia inhibitory factor
TGF-β: Transforming growth factor-beta
FGF: Fibroblast growth factor
MMP: Matrix metalloproteinase
miRNA: MicroRNA
GZMB: Granzyme B
CD8a: CD8a molecule
FFPE: Formalin-fixed paraffin-embedded
DMEM: Dulbecco’s Modified Eagle Medium
FBS: Fetal bovine serum
RPMI: Roswell Park Memorial Institute
STR: Short tandem repeat
NOD/SCID: Non-obese diabetic/severe-combined immunodeficient
cDNA: Complementary DNA
DEG: Differentially expressed gene
GSEA: Gene set enrichment analysis
KEGG: Kyoto encyclopedia of genes and genomes
BSA: Bovine serum albumin
TEM: Transmission electron microscopy
CD9: CD9 molecule
FN1: Fibronectin 1
IGFBP3: Insulin growth factor binding protein 3
TNC: Tenascin C
COL6A3: Collagen type VI alpha 3 chain
COL10A1: Collagen type X alpha 1 chain
COL12A1: Collagen type XII alpha 1 chain
RELB: RELB proto-oncogene
NOD: Nucleotide-binding oligomerization domain
TNF: Tumor necrosis factor
FoxO: Forkhead box O
IL17: Interleukin 17
HIF-1: Hypoxia-inducible factor 1
EMT: Epithelial-mesenchymal transition
qRT-PCR: Quantitative real-time reverse transcription polymerase chain reaction
PBS: Phosphate-buffered saline
EdU: 5-Ethynyl-2′-deoxyuridine
mTOR: Mammalian target of rapamycin
p70S6K1: Ribosomal protein S6 kinase beta-1
MEK1/2: Mitogen-activated protein kinase kinase 2
ERK1/2: Mitogen-activated protein kinase 3/1
p38 MAPK: p38 mitogen-activated protein kinase
ECM: Extracellular matrix
PDGFRα/β: Platelet-derived growth factor receptor α/β
CYR61: Cysteine rich angiogenic inducer 61
CTGF: Connective tissue growth factor
SRC: SRC proto-oncogene
IC50: Half maximal inhibitory concentration
ELISA: Enzyme-linked immunosorbent assay
CSF1: Colony stimulating factor 1
TCGA: The Cancer Genome Atlas
GZMH: Granzyme H
SHP2: Src homology region 2-containing protein tyrosine phosphatase 2
CBLB: Cbl proto-oncogene B
IL10: Interleukin 10
IL2RA: Interleukin 2 receptor subunit alpha
PPIA: Peptidylprolyl isomerase A
HSP70: Heat shock protein 70
GAPDH: Glyceraldehyde-3-phosphate dehydrogenase

## References

[1] De Wever O, Van Bockstal M, Mareel M, Hendrix A, Bracke M (2014). Carcinoma-associated fibroblasts provide operational flexibility in metastasis. Semin Cancer Biol.

[2] Ohlund D, Elyada E, Tuveson D (2014). Fibroblast heterogeneity in the cancer wound. J Exp Med.

[3] Yu Y, Ke L, Lv X, Ling YH, Lu J, Liang H, Qiu W, Huang X, Liu G, Li W (2018). The prognostic significance of carcinoma-associated fibroblasts and tumor-associated macrophages in nasopharyngeal carcinoma. Cancer Manag Res.

[4] Zhu YH, Shi C, Zeng L, Liu GZ, Jiang WH, Zhang X, Chen SL, Guo JJ, Jian XX, Ouyang J (2020). High COX-2 expression in cancer-associated fibiroblasts contributes to poor survival and promotes migration and invasiveness in nasopharyngeal carcinoma. Mol Carcinog.

[5] Wang S, Ma N, Kawanishi S, Hiraku Y, Oikawa S, Xie Y, Zhang Z, Huang G, Murata M (2014). Relationships of alpha-SMA-positive fibroblasts and SDF-1-positive tumor cells with neoangiogenesis in nasopharyngeal carcinoma. Biomed Res Int.

[6] Al-Nedawi K, Meehan B, Micallef J, Lhotak V, May L, Guha A, Rak J (2008). Intercellular transfer of the oncogenic receptor EGFRvIII by microvesicles derived from tumour cells. Nat Cell Biol.

[7] Kucharzewska P, Christianson HC, Welch JE, Svensson KJ, Fredlund E, Ringner M, Morgelin M, Bourseau-Guilmain E, Bengzon J, Belting M (2013). Exosomes reflect the hypoxic status of glioma cells and mediate hypoxia-dependent activation of vascular cells during tumor development. Proc Natl Acad Sci U S A.

[8] Park JE, Tan HS, Datta A, Lai RC, Zhang H, Meng W, Lim SK, Sze SK (2010). Hypoxic tumor cell modulates its microenvironment to enhance angiogenic and metastatic potential by secretion of proteins and exosomes. Mol Cell Proteomics.

[9] Zhou W, Fong MY, Min Y, Somlo G, Liu L, Palomares MR, Yu Y, Chow A, O'Connor ST, Chin AR (2014). Cancer-secreted miR-105 destroys vascular endothelial barriers to promote metastasis. Cancer Cell.

[10] Webber J, Steadman R, Mason MD, Tabi Z, Clayton A (2010). Cancer exosomes trigger fibroblast to myofibroblast differentiation. Cancer Res.

[11] Young LS, Rickinson AB (2004). EPSTEIN-BARR VIRUS: 40 YEARS ON. Nat Rev Cancer.

[12] Munz C, Moormann A (2008). Immune escape by Epstein-Barr virus associated malignancies. Semin Cancer Biol.

[13] Middeldorp JM, Pegtel DM (2008). Multiple roles of LMP1 in Epstein-Barr virus induced immune escape. Semin Cancer Biol.

[14] Pai S, Khanna R (2001). Role of LMP1 in immune control of EBV infection. Semin Cancer Biol.

[15] Lai HC, Hsiao JR, Chen CW, Wu SY, Lee CH, Su IJ, Takada K, Chang Y (2010). Endogenous latent membrane protein 1 in Epstein-Barr virus-infected nasopharyngeal carcinoma cells attracts T lymphocytes through upregulation of multiple chemokines. Virology.

[16] Li J, Qian CN, Zeng YX (2009). Regulatory T cells and EBV associated malignancies. Int Immunopharmacol.

[17] Kanegane H, Nomura K, Miyawaki T, Tosato G (2002). Biological aspects of Epstein-Barr virus (EBV)-infected lymphocytes in chronic active EBV infection and associated malignancies. Crit Rev Oncol Hematol.

[18] Ogino T, Moriai S, Ishida Y, Ishii H, Katayama A, Miyokawa N, Harabuchi Y, Ferrone S (2007). Association of immunoescape mechanisms with Epstein-Barr virus infection in nasopharyngeal carcinoma. Int J Cancer.

[19] Liu SC, Tsang NM, Chiang WC, Chang KP, Hsueh C, Liang Y, Juang JL, Chow KP, Chang YS (2013). Leukemia inhibitory factor promotes nasopharyngeal carcinoma progression and radioresistance. J Clin Invest.

[20] Zheng H, Li LL, Hu DS, Deng XY, Cao Y (2007). Role of Epstein-Barr virus encoded latent membrane protein 1 in the carcinogenesis of nasopharyngeal carcinoma. Cell Mol Immunol.

[21] Meckes DG, Shair KH, Marquitz AR, Kung CP, Edwards RH, Raab-Traub N (2010). Human tumor virus utilizes exosomes for intercellular communication. Proc Natl Acad Sci U S A.

[22] Canitano A, Venturi G, Borghi M, Ammendolia MG, Fais S (2013). Exosomes released in vitro from Epstein-Barr virus (EBV)-infected cells contain EBV-encoded latent phase mRNAs. Cancer Lett.

[23] Aga M, Bentz GL, Raffa S, Torrisi MR, Kondo S, Wakisaka N, Yoshizaki T, Pagano JS, Shackelford J (2014). Exosomal HIF1alpha supports invasive potential of nasopharyngeal carcinoma-associated LMP1-positive exosomes. Oncogene.

[24] Houali K, Wang X, Shimizu Y, Djennaoui D, Nicholls J, Fiorini S, Bouguermouh A, Ooka T (2007). A new diagnostic marker for secreted Epstein-Barr virus encoded LMP1 and BARF1 oncoproteins in the serum and saliva of patients with nasopharyngeal carcinoma. Clin Cancer Res.

[25] Klibi J, Niki T, Riedel A, Pioche-Durieu C, Souquere S, Rubinstein E, Le Moulec S, Guigay J, Hirashima M, Guemira F (2009). Blood diffusion and Th1-suppressive effects of galectin-9-containing exosomes released by Epstein-Barr virus-infected nasopharyngeal carcinoma cells. Blood.

[26] Huang SCM, Tsao SW, Tsang CM. Interplay of viral infection, host cell factors and tumor microenvironment in the pathogenesis of nasopharyngeal carcinoma. Cancers (Basel). 2018;10. 10.3390/cancers10040106 PMC5923361 29617291

[27] Tan GW, Visser L, Tan LP, van den Berg A, Diepstra A. The microenvironment in Epstein-Barr virus-associated malignancies. Pathogens. 2018;7. 10.3390/pathogens7020040 PMC6027429 29652813

[28] Yoshizaki T, Kondo S, Endo K, Nakanishi Y, Aga M, Kobayashi E, Hirai N, Sugimoto H, Hatano M, Ueno T (2018). Modulation of the tumor microenvironment by Epstein-Barr virus latent membrane protein 1 in nasopharyngeal carcinoma. Cancer Sci.

[29] Zhao B, Tumaneng K, Guan KL (2011). The hippo pathway in organ size control, tissue regeneration and stem cell self-renewal. Nat Cell Biol.

[30] Basu S, Totty NF, Irwin MS, Sudol M, Downward J (2003). Akt phosphorylates the yes-associated protein, YAP, to induce interaction with 14-3-3 and attenuation of p73-mediated apoptosis. Mol Cell.

[31] Kanai F, Marignani PA, Sarbassova D, Yagi R, Hall RA, Donowitz M, Hisaminato A, Fujiwara T, Ito Y, Cantley LC, Yaffe MB (2000). TAZ: a novel transcriptional co-activator regulated by interactions with 14-3-3 and PDZ domain proteins. EMBO J.

[32] Liu F, Lagares D, Choi KM, Stopfer L, Marinkovic A, Vrbanac V, Probst CK, Hiemer SE, Sisson TH, Horowitz JC (2015). Mechanosignaling through YAP and TAZ drives fibroblast activation and fibrosis. Am J Phys Lung Cell Mol Phys.

[33] Mannaerts I, Leite SB, Verhulst S, Claerhout S, Eysackers N, Thoen LF, Hoorens A, Reynaert H, Halder G, van Grunsven LA (2015). The hippo pathway effector YAP controls mouse hepatic stellate cell activation. J Hepatol.

[34] Lin CT, Chan WY, Chen W, Huang HM, Wu HC, Hsu MM, Chuang SM, Wang CC (1993). Characterization of seven newly established nasopharyngeal carcinoma cell lines. Lab Investig.

[35] Hänzelmann S, Castelo R, Guinney J (2013). GSVA: gene set variation analysis for microarray and RNA-Seq data. BMC Bioinformatics.

[36] Wu T, Hu E, Xu S, Chen M, Guo P, Dai Z, Feng T, Zhou L, Tang W, Zhan L, et al: clusterProfiler 4.0: A universal enrichment tool for interpreting omics data. Innovation (N Y) 2021, 2:100141. 10.1016/j.xinn.2021.100141 PMC8454663 34557778

[37] Subramanian A, Tamayo P, Mootha VK, Mukherjee S, Ebert BL, Gillette MA, Paulovich A, Pomeroy SL, Golub TR, Lander ES, Mesirov JP (2005). Gene set enrichment analysis: a knowledge-based approach for interpreting genome-wide expression profiles. Proc Natl Acad Sci U S A.

[38] Liu SC, Hsu T, Chang YS, Chung AK, Jiang SS, OuYang CN, et al. Cytoplasmic LIF reprograms invasive mode to enhance NPC dissemination through modulating YAP1-FAK/PXN signaling. Nat Commun. 2018;9. 10.1038/s41467-018-07660-6 PMC6269507 30504771

[39] Nardone G (2017). Oliver-De La Cruz J, Vrbsky J, martini C, Pribyl J, Skladal P, Pesl M, Caluori G, Pagliari S, Martino F, et al: YAP regulates cell mechanics by controlling focal adhesion assembly. Nat Commun.

[40] Elosegui-Artola A, Oria R, Chen Y, Kosmalska A, Perez-Gonzalez C, Castro N, Zhu C, Trepat X, Roca-Cusachs P (2016). Mechanical regulation of a molecular clutch defines force transmission and transduction in response to matrix rigidity. Nat Cell Biol.

[41] Zhao B, Li L, Wang L, Wang CY, Yu J, Guan KL (2012). Cell detachment activates the hippo pathway via cytoskeleton reorganization to induce anoikis. Genes Dev.

[42] Panciera T, Azzolin L, Cordenonsi M, Piccolo S (2017). Mechanobiology of YAP and TAZ in physiology and disease. Nat Rev Mol Cell Biol.

[43] Ramjee V, Li D, Manderfield LJ, Liu F, Engleka KA, Aghajanian H, Rodell CB, Lu W, Ho V, Wang T (2017). Epicardial YAP/TAZ orchestrate an immunosuppressive response following myocardial infarction. J Clin Invest.

[44] Noguchi S, Saito A, Nagase T. YAP/TAZ signaling as a molecular link between fibrosis and Cancer. Int J Mol Sci. 2018;19. 10.3390/ijms19113674 PMC6274979 30463366

[45] Chang YM, Bai L, Liu S, Yang JC, Kung HJ, Evans CP (2008). Src family kinase oncogenic potential and pathways in prostate cancer as revealed by AZD0530. Oncogene.

[46] Brennen WN, Isaacs JT, Denmeade SR (2012). Rationale behind targeting fibroblast activation protein-expressing carcinoma-associated fibroblasts as a novel chemotherapeutic strategy. Mol Cancer Ther.

[47] Moroishi T, Hansen CG, Guan KL (2015). The emerging roles of YAP and TAZ in cancer. Nat Rev Cancer.

[48] Chen YP, Yin JH, Li WF, Li HJ, Chen DP, Zhang CJ, Lv JW, Wang YQ, Li XM, Li JY (2020). Single-cell transcriptomics reveals regulators underlying immune cell diversity and immune subtypes associated with prognosis in nasopharyngeal carcinoma. Cell Res.

