# Supplementary material for: Epstein-Barr viral product-containing exosomes promote fibrosis and nasopharyngeal carcinoma progression through activation of YAP1/FAPα signaling in fibroblasts
Source: J Exp Clin Cancer Res. 2022 Aug 20;41:254. doi: 10.1186/s13046-022-02456-5 (PMC9392321; doi:10.1186/s13046-022-02456-5)
Supplement: Supplementary file 1 — Additional file 1: Supplementary Table S1. Baseline Characteristics of the YAP1 Study Population. [file 13046_2022_2456_MOESM1_ESM.pdf]

Supplementary Table S1. Baseline Characteristics of the YAP1 Study Population

YAP1 Score	<80	≥80	Total	p value
Number (n=106)	55 (51.9%)	51 (48.1%)	106 (100%)	
YAP1 (median)	40 (0-70)	100 (80-230)	70 (0-230)	<0.001[a]
Mean (± SD)	31.59±24.45	105.14±38.91	71.5±53.11	
Age (years) Median	47.87 (26.11-68.66)	48.01 (20.96-81.35)	47.93 (20.96-81.35)	0.500[a]
Mean (± SD)	48.44±9.62	49.97±13.21	49.18±11.47	
Total Treatment Days	54.0 (44-65)	53.0 (44-64)	53.0 (44-65)	0.534[a]
Mean (± SD)	53.89±4.33	53.37±4.20	53.64±4.26	
BED (median)	86.4 (79.2-91.2)	86.4 (74.3-91.2)	86.4 (74.34-91.2)	0.275[a]
Mean (± SD)	85.35±2.87	86.02±3.36	85.68±3.12	
Gender				0.280[b]
Female	12 (21.8%)	16 (31.4%)	28 (26.4%)	
Male	43 (78.2%)	35 (68.6%)	78 (73.6%)	
Age				0.846[b]
<48	29 (52.7%)	25 (49.0%)	54 (50.9%)	
≥48	26 (47.3%)	26 (51.0%)	52 (49.1%)	
AJCC 2010 T-Stage				1[b]
$T_{1/2}$	28 (50.9%)	25 (49.0%)	53 (50%)	
$T_{3/4}$	27 (49.1%)	26 (51.0%)	53 (50%)	
AJCC 2010 N-Stage				0.443[b]
$N_{0/1}$	27 (49.1%)	29 (56.9%)	56 (52.8%)	
$N_{2/3}$	28 (50.9%)	22 (43.1%)	50 (47.2%)	
Chemotherapy				1[b]
Yes	42 (76.4%)	38 (74.5%)	80 (75.5%)	
No	13 (23.6%)	13 (25.5%)	26 (24.5%)	
Comorbidity				1[b]
No	34 (61.8%)	32 (62.7%)	66 (62.3%)	
Yes	21 (38.2%)	19 (37.3%)	40 (37.7%)	
Smoking				
No	25 (45.5%)	23 (45.1%)	48 (45.3%)	1[b]
Yes	30 (54.5%)	28 (54.9%)	58 (54.7%)	
Betel Quid				0.633[b]
No	45 (81.8%)	39 (76.5%)	84 (79.2%)	
Yes	10 (18.2%)	12 (23.5%)	22 (20.8%)	
Alcohol				0.423[b]
No	37 (67.3%)	30 (58.8%)	67 (63.2%)	
Yes	18 (32.7%)	21 (41.2%)	39 (36.8%)	
OS (years) Median	10.74 (0.16-18.24)	10.05 (0.42-16.01)	10.41 (0.16-18.24)	0.058[a]
Mean (± SD)	10.06±4.62	8.32±4.69	9.22±4.71	

[a]p-value was calculated using ANOVA test, 2-tailed. [b]Obtained using chi-square test, 2-tailed.
BED: biologically effective dose; OS: overall survival.
