# Supplementary material for: Epstein-Barr viral product-containing exosomes promote fibrosis and nasopharyngeal carcinoma progression through activation of YAP1/FAPα signaling in fibroblasts
Source: J Exp Clin Cancer Res. 2022 Aug 20;41:254. doi: 10.1186/s13046-022-02456-5 (PMC9392321; doi:10.1186/s13046-022-02456-5)
Supplement: Supplementary file 2 — Additional file 2: Supplementary Table S2. Baseline Characteristics of the FAPα Study Population. [file 13046_2022_2456_MOESM2_ESM.pdf]

Supplementary Table S2. Baseline Characteristics of the FAPα Study Population

FAPα Score	<80	≥80	Total	p value
Number (n=143)	90 (62.9%)	53 (37.1%)	143 (100%)	
FAPα (median)	20 (0-75)	100 (80-230)	50 (0-230)	<0.001[a]
Mean (± SD)	25.44±23.57	111.32±34.08	57.27±50.06	
Age (years) Median	47.96 (21.48-73.84)	50.37 (26.65-81.35)	48.96 (21.48-81.35)	0.213[a]
Mean (± SD)	48.44±10.11	50.87±11.71	49.34±10.75	
Total Treatment days	54.0 (44-69)	55.0 (45-70)	54.0 (44-70)	0.096[a]
Mean (± SD)	54.06±4.90	55.49±4.96	54.59±4.96	
BED (median)	86.4 (79.2-107.36)	87.12 (74.34-105.46)	86.4 (74.34-107.36)	0.013[a]
Mean (± SD)	85.13±4.11	87.12±4.80	85.87±4.47	
Gender				0.443[b]
Female	23 (25.6%)	17 (32.1%)	40 (28.0%)	
Male	67 (74.4%)	36 (67.9%)	103 (72.0%)	
Age				
<48	45 (50.0%)	23 (43.4%)	68 (47.6%)	0.491[b]
≥48	45 (50.0%)	30 (56.6%)	75 (52.4%)	
AJCC 2010 T-Stage				0.002[b]
$T_{1/2}$	52 (57.8%)	16 (30.2%)	68 (47.6%)	
$T_{3/4}$	38 (42.2%)	37 (69.8%)	75 (52.4%)	
AJCC 2010 N-Stage				0.604[b]
$N_{0/1}$	44 (48.9%)	29 (54.7%)	73 (51.0%)	
$N_{2/3}$	46 (51.1%)	24 (45.3%)	70 (49.0%)	
Chemotherapy				0.208[b]
Yes	68 (75.6%)	45 (84.9%)	113 (79.0%)	
No	22 (24.4%)	8 (15.1%)	30 (21.0%)	
Comorbidity				0.591[b]
No	59 (65.6%)	32 (60.4%)	91 (63.6%)	
Yes	31 (34.4%)	21 (39.6%)	52 (36.4%)	
Smoking				0.728[b]
No	41 (45.6%)	22 (41.5%)	63 (44.1%)	
Yes	49 (54.4%)	31 (58.5%)	80 (55.9%)	
Betel Quid				0.535[b]
No	68 (75.6%)	43 (81.1%)	111 (77.6%)	
Yes	22 (24.4%)	10 (18.9%)	32 (22.4%)	
Alcohol				0.091[b]
No	59 (65.6%)	42 (79.2%)	101 (70.6%)	
Yes	31 (34.4%)	11 (20.8%)	42 (29.4%)	
OS (years) Median	9.64 (0.16-18.93)	8.03 (0.42-18.06)	8.53 (0.16-18.93)	0.032[a]
Mean (± SD)	9.77±5.05	8.04±4.34	9.13±4.85	

[a]p-value was calculated using ANOVA test, 2-tailed. [b]Obtained using chi-square test, 2-tailed.
BED: biologically effective dose; OS: overall survival.
