# Supplementary material for: Epstein-Barr viral product-containing exosomes promote fibrosis and nasopharyngeal carcinoma progression through activation of YAP1/FAPα signaling in fibroblasts
Source: J Exp Clin Cancer Res. 2022 Aug 20;41:254. doi: 10.1186/s13046-022-02456-5 (PMC9392321; doi:10.1186/s13046-022-02456-5)
Supplement: Supplementary file 3 — Additional file 3: Supplementary Table S3. Primer information for quantitative RT-PCR analysis. [file 13046_2022_2456_MOESM3_ESM.pdf]

Supplementary Table S3. Primer information for quantitative RT-PCR analysis

Gene Symbol	Gene Product	Forward primer sequence (5'-3')	Reverse Primeer sequence (5'-3')
CCL2	C-C motif chemokine ligand 2	CTGAGACTAACCCAGAAACATC	GAATGAAGGTGGCTGCTAT
COL1A1	Collagen type I alpha 1 chain	CCTGGAAAGAATGGAGATGAT	CCACTGAAACCTCTGTGTC
CSF1	Colony stimulating factor 1	GAATGCTCCAGCCAAGA	AGAGTCCTCCCAGGTCAA
CTGF	Connective tissue growth factor; Cellular communication network factor 2	AAGAGAACATTAAGAAGGGCAA	TCCACAGAATTTAGCTCGGTA
CYR61	Cysteine-rich angiogenic inducer 61; Cellular communication network factor 1	GAATGGAGCCTCGCATC	CACAGGTCTTTGAGCACT
FAPα	Fibroblast activation protein alpha; Prolyl endopeptidase FAP	CGTGGGTTACTGATGAACGA	TCCTGGGTCTTTGGACAAT
FGF2	Fibroblast growth factor 2	GCAGAAGAGAGAGGAGTTG	TCATCCGTAACACATTTAGAAGC
FN1	Fibronectin 1	GATTCAGAGACTGGGACG	CTTGGATAGGTCTGTAAAGGTT
GAPDH	Glyceraldehyde-3-phosphate dehydrogenase	TCCTCTGACTTCAACAGCGA	TCCTTGGAGGCCATGTG
IGF1	Insulin like growth factor 1	CCTTTCAAGCCACCCAT	AGTGTGTTTAGCAGCGG
IGF1R	Insulin-like growth factor 1 receptor	ATGCTTCAGAAACACCTCAATA	TGGATAGATGATGCCTTGGTA
IGFBP3	Insulin-like growth factor-binding protein 3	TAAGTATGGGCAGCCTCTC	ATGTCCTTGGCAGTCTTT
IL1A	Interleukin-1 alpha	CACGGCACTAAGAACTATTT	TGAGACTCCAGACCTACG
IL1R1	Interleukin-1 receptor type 1	ACGGTCACCTTCATCTAAACA	CATAAGACAGGAGGCACCTA
IL4R	Interleukin 4 receptor	CAGAGAGCATCAGCGTG	TCCCTCCTGGAAGTCAT
IL6	Interleukin 6	GTACCTCCAGAACAGATTTGAGA	TGGCATTTGTGGTTGGG
IL8	Interleukin 8	CCAAGAATCAGTGAAGATGC	GCAACCCTACAACAGACC
LAMA1	Laminin subunit alpha 1	CCACTGTGCTCTGTGATG	CACTGAGGTAGACTGGGT
PDGFA	Platelet derived growth factor subunit A	GATGAGATGGAGGGTCGC	CTCTGGAGTCGTTCCCAA
PDGFB	Platelet derived growth factor subunit B	ACAAGACGGCACTGAAG	GAGACAGACGGACGAGG
PDGFRA	Platelet derived growth factor receptor alpha	GATGAGATGGAGGGTCGC	CTCTGGAGTCGTTCCCAA
PPIA	Peptidylprolyl isomerase A	TGCTGACTGTGGACAAC	AGGATACTGCGAGCAAAT
RARRES2	Retinoic acid receptor responder 2	GAGTGCGAACTGACTGAT	GTGAAGGAGCCTGGAAATG
SMAD3	SMAD family member 3	CTCTCCAATGTCAACAGGAAT	CTGAGGCACTCTGCGAA
VEGFA	Vascular endothelial growth factor A	GGCTCTGACCAGGAGTT	CTGAATCTTCCAGGCAGTG
VEGFR	Vascular endothelial growth factor receptor 1	GTGTCAGAATCCCTGCGA	TGTCTCTTTCACTCACTTCCATA
YAP1	Yes-associated protein 1	AGCTTCCTTTGTCCAGTG	CAGTCTTCAGCCTTGGTT
