# Supplementary figures and images for: Epstein-Barr viral product-containing exosomes promote fibrosis and nasopharyngeal carcinoma progression through activation of YAP1/FAPα signaling in fibroblasts
Source: J Exp Clin Cancer Res. 2022 Aug 20;41:254. doi: 10.1186/s13046-022-02456-5 (PMC9392321; doi:10.1186/s13046-022-02456-5)

Supplementary Figure S1

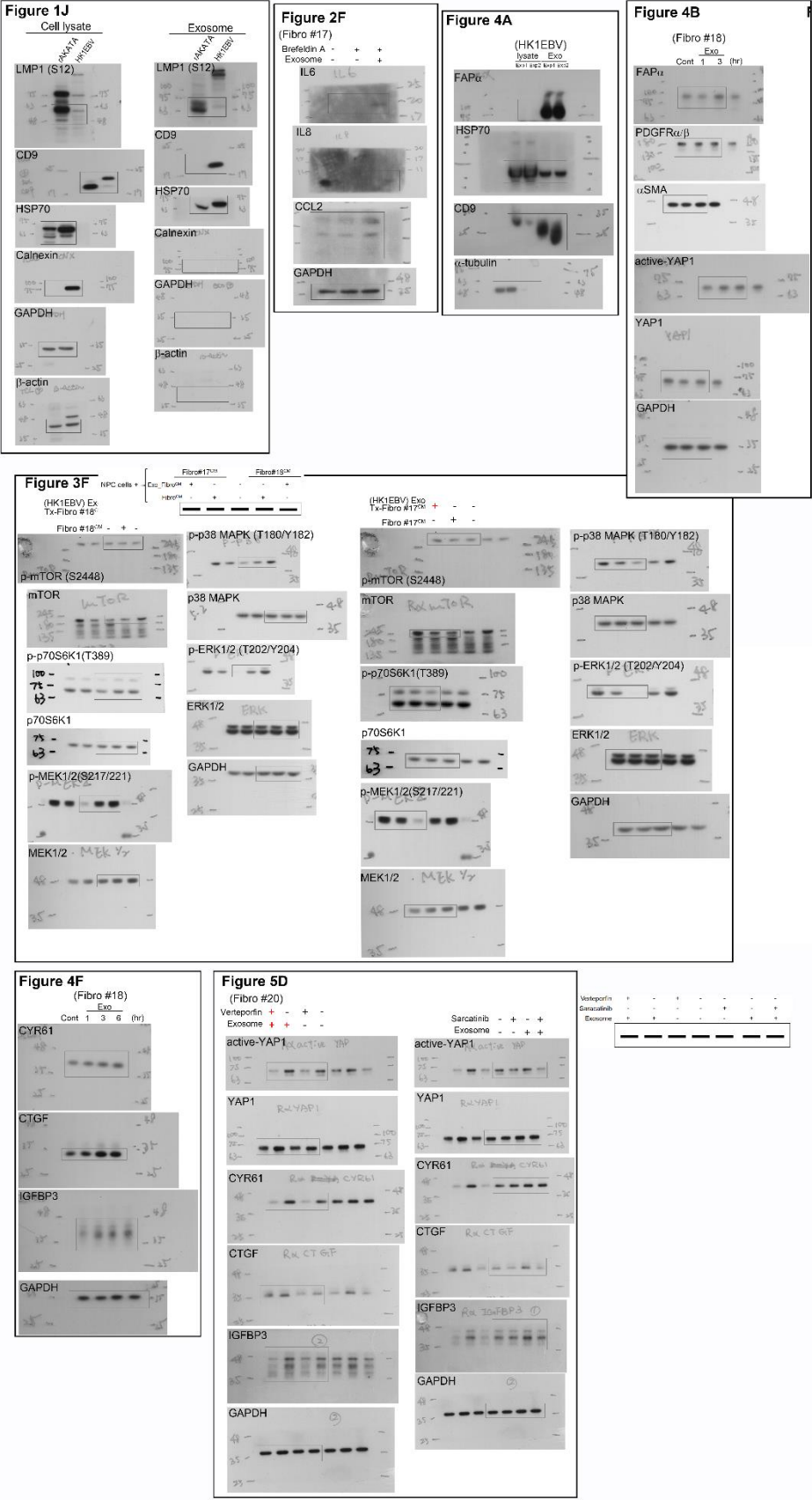

Supplementary Figure S1. Uncropped western blots for figures.

Supplement: Supplementary file 4 — Additional file 4: Supplementary Fig. S1. Uncropped western blots for figures. [file 13046_2022_2456_MOESM4_ESM.pdf]
