# Supplementary material for: Epstein-Barr viral product-containing exosomes promote fibrosis and nasopharyngeal carcinoma progression through activation of YAP1/FAPα signaling in fibroblasts
Source: J Exp Clin Cancer Res. 2022 Aug 20;41:254. doi: 10.1186/s13046-022-02456-5 (PMC9392321; doi:10.1186/s13046-022-02456-5)
Supplement: Supplementary file 6 — Additional file 6: Supplementary Fig. S3. Uptake of exosomes by primary fibroblasts. Exosomes were pre-labeled with green fluorescent dye and added to cultures of fibroblasts. Images were captured 24 hours post exosome stimulation. Scale bar, 50 μm. [file 13046_2022_2456_MOESM6_ESM.pdf]

Supplementary Figure S3

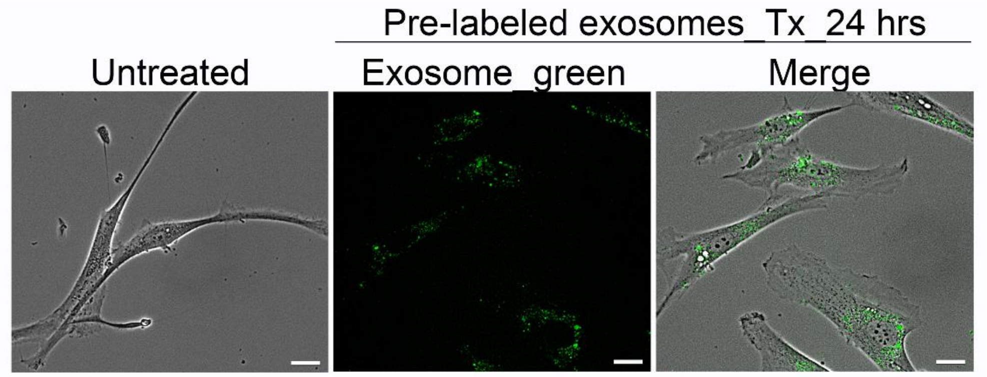

Supplementary Figure S3. Uptake of exosomes by primary fibroblasts. Exosomes were pre-labeled with green fluorescent dye and added to cultures of fibroblasts. Images were captured 24 hours post exosome stimulation. Scale bar, 50 μm.
