# Supplementary material for: Epstein-Barr viral product-containing exosomes promote fibrosis and nasopharyngeal carcinoma progression through activation of YAP1/FAPα signaling in fibroblasts
Source: J Exp Clin Cancer Res. 2022 Aug 20;41:254. doi: 10.1186/s13046-022-02456-5 (PMC9392321; doi:10.1186/s13046-022-02456-5)
Supplement: Supplementary file 7 — Additional file 7: Supplementary Fig. S4. Representative IHC images of PDGFRα/β, active YAP1, FAPα, and trichrome staining in paraffin-embedded consecutive NPC xenografts. Black arrows signify fibroblast-like cellular structures. Yellow marked zones indicate selected tumor beds. Fibrosis (blue) within tumor sections was evaluated using trichrome staining. Scale bar, 20 μm. [file 13046_2022_2456_MOESM7_ESM.pdf]

Supplementary Figure S4

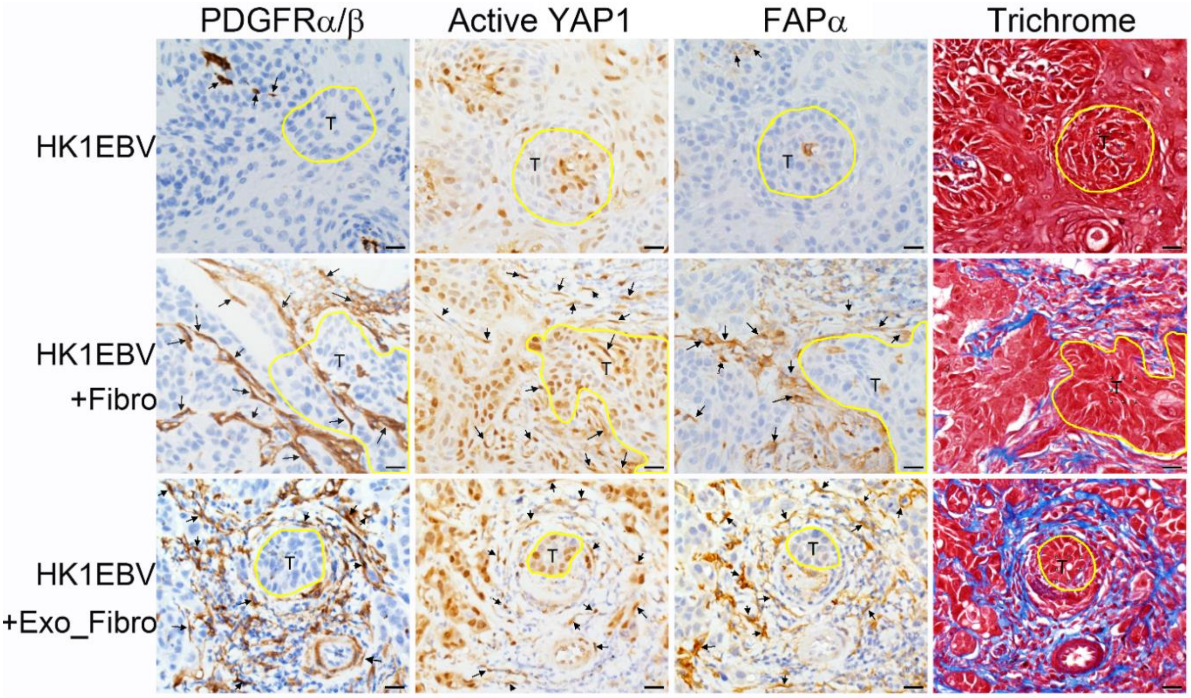

Supplementary Figure S4. Representative IHC images of PDGFRα/β, active YAP1, FAPα, and trichrome staining in paraffin-embedded consecutive NPC xenografts. Black arrows signify fibroblast-like cellular structures. Yellow marked zones indicate selected tumor beds. Fibrosis (blue) within tumor sections was evaluated using trichrome staining. Scale bar, 20 μm.
