# Supplementary material for: Epstein-Barr viral product-containing exosomes promote fibrosis and nasopharyngeal carcinoma progression through activation of YAP1/FAPα signaling in fibroblasts
Source: J Exp Clin Cancer Res. 2022 Aug 20;41:254. doi: 10.1186/s13046-022-02456-5 (PMC9392321; doi:10.1186/s13046-022-02456-5)
Supplement: Supplementary file 8 — Additional file 8: Supplementary Fig. S5. Images of xenografts dissected from NOD/SCID mice after 7-week subcutaneous injection. HK1EBV cells, HK1EBV cells together with fibroblasts, or HK1EBV cells together with exosome-treated fibroblasts were subcutaneously injected into the legs of mice (n = 8–9 mice/group). [file 13046_2022_2456_MOESM8_ESM.pdf]

Supplementary Figure S5

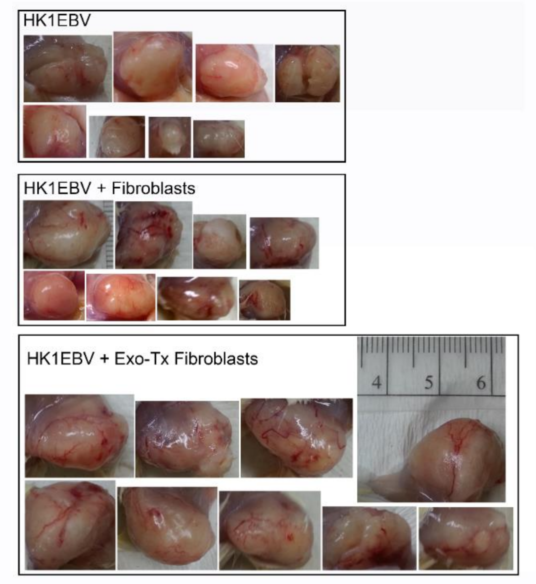

Supplementary Figure S5. Images of xenografts dissected from NOD/SCID mice after 7-week subcutaneous injection. HK1EBV cells, HK1EBV cells together with fibroblasts, or HK1EBV cells together with exosome-treated fibroblasts were subcutaneously injected into the legs of mice (n = 8–9 mice/group).
