# Supplementary material for: Epstein-Barr viral product-containing exosomes promote fibrosis and nasopharyngeal carcinoma progression through activation of YAP1/FAPα signaling in fibroblasts
Source: J Exp Clin Cancer Res. 2022 Aug 20;41:254. doi: 10.1186/s13046-022-02456-5 (PMC9392321; doi:10.1186/s13046-022-02456-5)
Supplement: Supplementary file 9 — Additional file 9: Supplementary Fig. S6. Deregulated signaling pathways in fibroblasts treated with EBV product-containing exosomes. Analysis of signaling pathways in fibroblasts stimulated with HK1EBV cell-derived exosomes compared with those exposed to medium. The top-scoring signaling pathways were identified using Ingenuity Pathway Analysis (IPA) software (QIAGEN). The identified pathways were ranked by significance [−log (p-value)]. The number of genes that met cutoff criteria [|log2(FC)| > 0.5 and p value < 0.05] in a given signaling pathway was shown. Total RNAs were harvested 6 hours post HK1EBV cell-derived exosome (10 μg/ml) stimulation. [file 13046_2022_2456_MOESM9_ESM.pdf]

Supplementary Figure S6

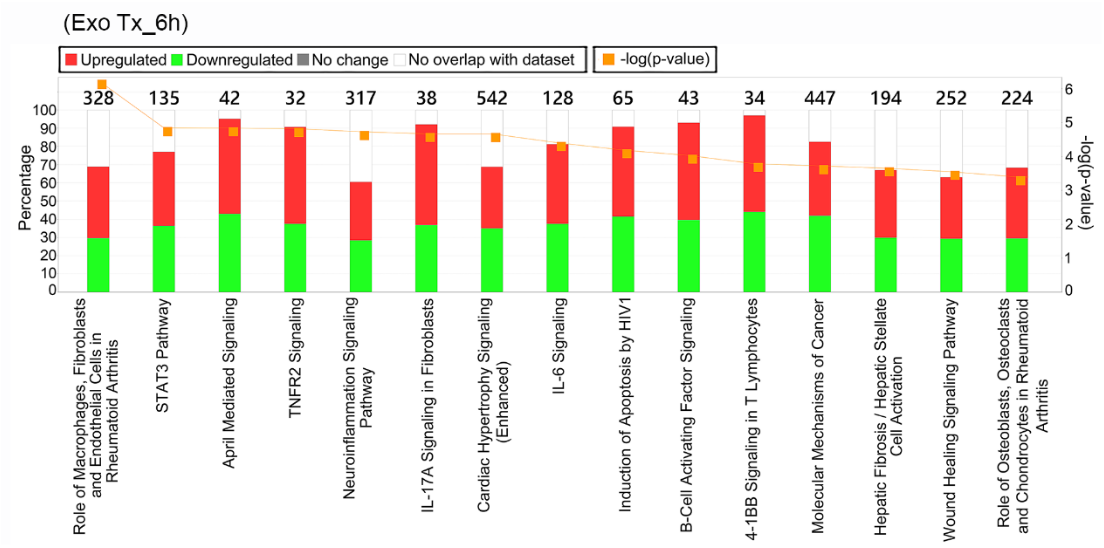

Supplementary Figure S6. Deregulated signaling pathways in fibroblasts treated with EBV product-containing exosomes. Analysis of signaling pathways in fibroblasts stimulated with HK1EBV cell-derived exosomes compared with those exposed to medium. The top-scoring signaling pathways were identified using Ingenuity Pathway Analysis (IPA) software (QIAGEN). The identified pathways were ranked by significance [-log (p-value)]. The number of genes that met cutoff criteria [$|log2(FC)| > 0.5$ and p value < 0.05] in a given signaling pathway was shown. Total RNAs were harvested 6 hours post HK1EBV cell-derived exosome (10 μg/ml) stimulation.
