# Supplementary material for: Epstein-Barr viral product-containing exosomes promote fibrosis and nasopharyngeal carcinoma progression through activation of YAP1/FAPα signaling in fibroblasts
Source: J Exp Clin Cancer Res. 2022 Aug 20;41:254. doi: 10.1186/s13046-022-02456-5 (PMC9392321; doi:10.1186/s13046-022-02456-5)
Supplement: Supplementary file 10 — Additional file 10: Supplementary Fig. S7. Cytotoxicity assessments of different doses of verteporfin or saracatinib in primary fibroblasts. A–B Real-time measurement of cell survival in response to various doses of verteporfin (A) or saracatinib (B) treatment in Fibro#17 cells. C–D Measurement of cell survival in response to various doses of verteporfin (C) or saracatinib (D) treatment in Fibro#18 cells. Data are presented as means and SD of quadruplicate experiments (left panels). The drug-induced cell toxicity was determined by calculating the IC50 after treatment (right panels). Normalized cell growth was displayed against the logarithm of concentration. The values of IC50 were calculated using the RTCA software (ACEA Biosciences). [file 13046_2022_2456_MOESM10_ESM.pdf]

## Supplementary Figure S7

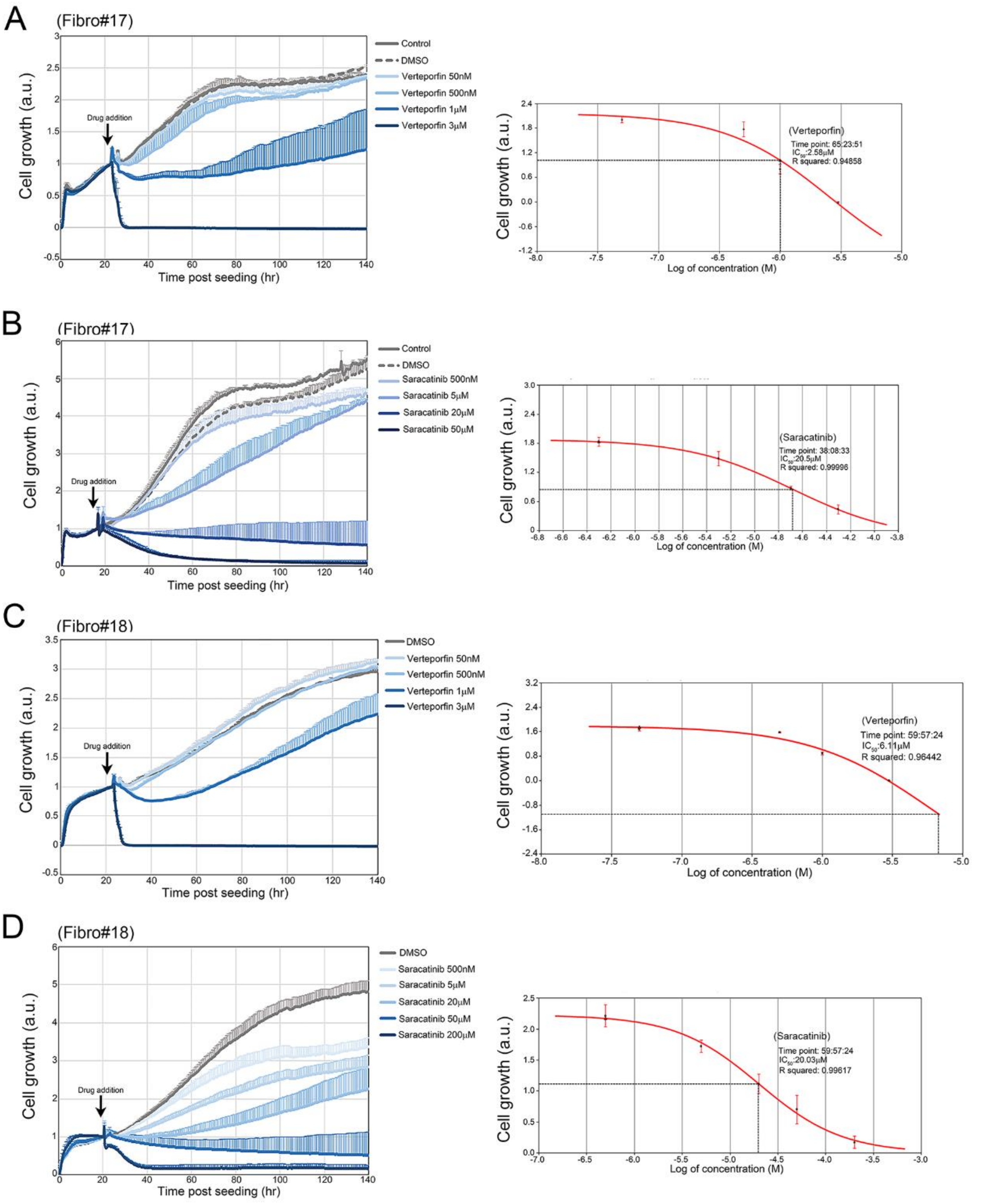


**Supplementary Figure S7.** Cytotoxicity assessments of different doses of verteporfin or saracatinib in primary fibroblasts. **A–B** Real-time measurement of cell survival in response to various doses of verteporfin (**A**) or saracatinib (**B)** treatment in Fibro#17 cells. **C–D** Measurement of cell survival in response to various doses of verteporfin (**C**)

or saracatinib (**D**) treatment in Fibro#18 cells. Data are presented as means and SD of quadruplicate experiments (left panels). The drug-induced cell toxicity was determined by calculating the $IC_{50}$ after treatment (right panels). Normalized cell growth was displayed against the logarithm of concentration. The values of $IC_{50}$ were calculated using the RTCA software (ACEA Biosciences).
