# Supplementary material for: Epstein-Barr viral product-containing exosomes promote fibrosis and nasopharyngeal carcinoma progression through activation of YAP1/FAPα signaling in fibroblasts
Source: J Exp Clin Cancer Res. 2022 Aug 20;41:254. doi: 10.1186/s13046-022-02456-5 (PMC9392321; doi:10.1186/s13046-022-02456-5)
Supplement: Supplementary file 11 — Additional file 11: Supplementary Fig. S8. Correlation analysis between expression of FAPα and immune regulatory molecules. Expression data were extracted from the TCGA head and neck squamous cell carcinoma RNA sequencing dataset. Spearman’s correlation test was used to calculate the correlation between mRNA expressions of two select genes. [file 13046_2022_2456_MOESM11_ESM.pdf]

Supplementary Figure S8

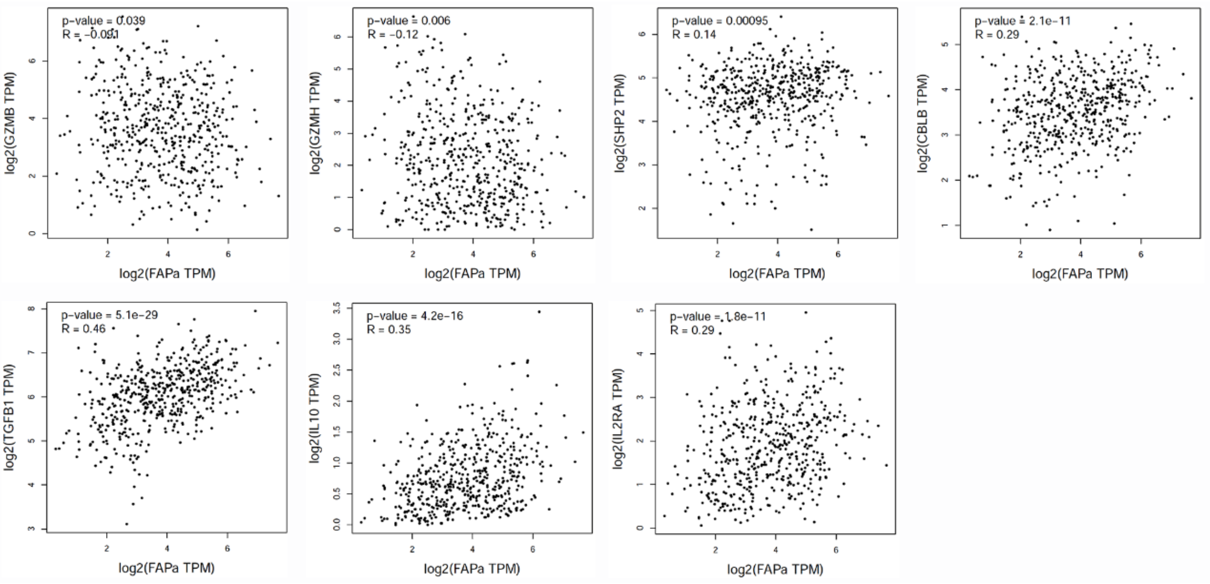

Supplementary Figure S8. Correlation analysis between expression of FAPα and immune regulatory molecules. Expression data were extracted from the *TCGA head and neck squamous cell carcinoma RNA sequencing dataset.* Spearman's correlation test was used to calculate the correlation between mRNA expressions of two select genes.
